# Keratinized Mucosa Width and Incidence of Peri‐Implant Diseases: A Systematic Review With Meta‐Analysis

**DOI:** 10.1111/jre.70123

**Published:** 2026-05-14

**Authors:** Isabella Neme Ribeiro dos Reis, Daniel Stefan Thoma, Thaís Emília da Silva, Milena Quesada Passos, Luisa Souza Feitosa, Cláudio Mendes Pannuti, Ronald Ernest Jung, Nadja Naenni

**Affiliations:** ^1^ Department of Reconstructive Dentistry, Center of Dental Medicine University of Zurich Zurich Switzerland; ^2^ Department of Stomatology, Division of Periodontics, School of Dentistry University of São Paulo São Paulo Brazil

**Keywords:** cohort studies, incidence, keratinized mucosa, meta‐analysis, peri‐implant mucositis, peri‐implantitis, systematic review

## Abstract

**Aim:**

To assess whether the presence and width of keratinized mucosa (KM) influence the *incidence* of peri‐implant mucositis and peri‐implantitis at the implant level.

**Methods:**

Electronic searches were conducted in MEDLINE/PubMed, Scopus, Embase, and Web of Science, complemented by gray literature and manual searches. Prospective and retrospective cohort studies reporting implant‐level incidence of peri‐implant diseases in sites with adequate versus inadequate KM were included. Random‐effects meta‐analyses were performed for two KM thresholds (> 0 mm vs. 0 mm; ≥ 2 mm vs. < 2 mm), and subgroup analyses compared prospective versus retrospective cohorts. Risk of bias assessment was performed using the Joanna Briggs Institute (JBI) checklist for cohort studies.

**Results:**

Twelve cohort studies (938 patients; 2646 implants) were included. KM was not associated with the incidence of peri‐implant mucositis for either threshold (> 0 mm vs. 0 mm: RR = 0.92, 95% CI 0.69–1.21; ≥ 2 mm vs. < 2 mm: RR = 0.70, 95% CI 0.41–1.20). In contrast, peri‐implantitis incidence was significantly lower in implants with KM (> 0 mm) compared to those without KM (0 mm) (RR = 0.62, 95% CI 0.42–0.91) and in implants with KM ≥ 2 mm compared to < 2 mm (RR = 0.56, 95% CI 0.37–0.84). No significant subgroup differences were observed between prospective and retrospective studies. According to the JBI checklist, 75% of the included studies were classified as having a low risk of bias.

**Conclusion:**

Evidence from cohort studies suggests no association between KM and peri‐implant mucositis incidence, whereas both the presence of KM and a width of ≥ 2 mm are associated with a lower incidence of peri‐implantitis. Thus, KM assessment may be considered as a part of peri‐implant risk evaluation.

## Graphical abstract

This systematic review and meta‐analysis of 12 cohort studies (2646 implants), the first restricted to cohort studies on this topic, found no association between keratinized mucosa and peri‐implant mucositis, but lower peri‐implantitis incidence in implants with keratinized mucosa (> 0 mm) and width ≥ 2 mm, supporting its consideration in risk assessment.

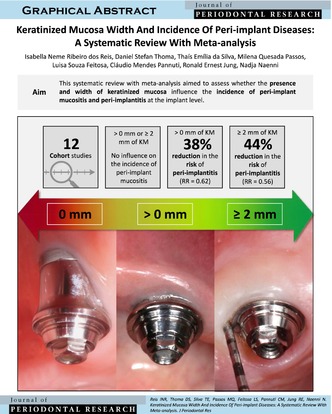

## 1 Introduction

The global increase in dental implant placement has been accompanied by a rising prevalence of peri‐implant diseases, which has become a significant public health concern [1]. Although implant therapy is in general characterized by high survival rates and patient satisfaction, the management of peri‐implant diseases, particularly peri‐implantitis, characterized by inflammation and progressive bone loss, remains challenging due to the limited predictability and effectiveness of current treatments [2, 3].

Given that disease resolution is often difficult to achieve, prevention is widely recognized as the most effective strategy for long‐term peri‐implant health [3, 4, 5]. Preventive approaches rely on the strict control of modifiable risk factors, making it essential to identify specific clinical elements that may act as protective factors [6, 7]. Such knowledge enables clinicians to move beyond generalized protocols and implement personalized maintenance strategies tailored to individual patient risk profiles.

An adequate band of keratinized mucosa (KM) has been widely proposed as one of these potential protective factors. KM is supposed to contribute to a stable biological seal around implants, protecting peri‐implant tissues from bacterial challenges and mechanical trauma [8]. In this context, increasing the width of keratinized mucosa through free gingival grafts has been associated with more favorable peri‐implant health outcomes, including lower bleeding indices and more stable radiographic bone levels [9, 10]. However, evidence that the absence or insufficient width of KM is associated with an increased risk of peri‐implant disease, thereby justifying soft tissue augmentation, remains inconclusive [11, 12, 13, 14].

While some systematic reviews report that a reduced width of KM (< 2 mm) is associated with increased plaque accumulation, inflammation, and marginal bone loss, others find limited or no evidence of its clinical relevance [11, 12, 15]. Most of the evidence synthesized by previous systematic reviews was derived from cross‐sectional studies (prevalence data) or focused on isolated clinical parameters, precluding temporal inferences regarding disease development. Therefore, to address these methodological gaps, this systematic review and meta‐analysis was restricted to longitudinal cohort studies, aiming to evaluate the effect of keratinized mucosa width on the actual incidence of peri‐implant diseases. We hypothesized that the absence or insufficient width of keratinized mucosa would be associated with an increased incidence of peri‐implant mucositis and peri‐implantitis.

## 2 Methods

### 2.1 Study Protocol and Registration

The protocol for this systematic review and meta‐analysis was registered in the PROSPERO database (CRD42024626459). The study was reported following the 2020 PRISMA guidelines for systematic reviews and meta‐analyses [16].

### 2.2 PECOS Question

The focused question of this systematic review was structured according to the PECOS framework: *What is the incidence of peri‐implant diseases in patients with dental implants with inadequate keratinized mucosa compared to those with adequate keratinized mucosa based on cohort studies?*

- *Population (P)*: Patients with dental implants.
- *Exposure (E)*: Inadequate keratinized mucosa width (e.g., ≤ 2 mm or absence of keratinized mucosa).
- *Comparison (C)*: Adequate width of keratinized mucosa.
- *Outcome (O)*: Incidence of peri‐implant diseases (peri‐implant mucositis and/or peri‐implantitis).
- *Study design (S)*: Prospective and retrospective cohort studies.

### 2.3 Inclusion and Exclusion Criteria

#### 2.3.1 Inclusion Criteria

- Prospective and retrospective cohort studies.
- Studies reporting both adequate and inadequate keratinized mucosa width and the incidence of peri‐implant diseases.
- Studies reporting the incidence of peri‐implant diseases in implants with adequate and inadequate keratinized mucosa.
- Participants aged 18 years or older.

#### 2.3.2 Exclusion Criteria

- Studies that did not include a diagnosis of peri‐implant diseases.
- Studies involving implants with machined surfaces.
- Studies that did not assess the incidence of peri‐implant diseases in areas with or without keratinized mucosa.

To account for the diversity of case definitions in the literature, the following criteria were used for the diagnosis of peri‐implant diseases during study selection:

- For peri‐implant mucositis, studies were eligible if they applied a diagnostic threshold based on clinical signs of inflammation, specifically bleeding on probing (BOP).
- For peri‐implantitis, studies were considered eligible if the diagnosis was based on longitudinal progressive bone loss, regardless of the specific radiographic cut‐off values applied (e.g., > 0.5, ≥ 1, ≥ 2, or ≥ 3 mm).

### 2.4 Search Strategies

To identify relevant studies, a comprehensive electronic search was conducted in four databases: the National Library of Medicine (MEDLINE/PubMed), SCOPUS, EMBASE, and Web of Science. The searches were performed from January 27, 2025 to October 31, 2025, with no restrictions regarding publication date or language. The complete search strategies used for each database are presented in Table S1.

In addition, gray literature was searched using OpenGrey and the Gray Literature Report databases. A manual search of the reference lists of all included studies was also carried out to identify potentially relevant articles that were not retrieved through the electronic database searches.

### 2.5 Selection Process

Following the electronic searches, all retrieved records were subjected to a multi‐step screening process carried out independently by two reviewers (MQP and TES). Initially, titles and abstracts were screened using the Rayyan web‐based tool to determine eligibility based on the predefined inclusion and exclusion criteria. Studies considered potentially eligible or those for which eligibility could not be clearly established from the title and abstract were selected for full‐text assessment.

In the subsequent phase, the full texts of the selected articles were carefully evaluated. Only studies that fulfilled all eligibility criteria were included for data extraction. Inter‐reviewer agreement was calculated using the kappa statistic. Any disagreements during the screening stages were resolved through discussion. When consensus could not be reached, a third and fourth reviewer (INRR and NN) were consulted to make the final inclusion decision.

### 2.6 Data Collection

Relevant information was independently collected from all eligible studies by three reviewers (MQP, TES, and LSF). The extracted variables included authorship and year of publication, study design, study setting, country, duration of follow‐up, sample size and distribution per group, number of implants per group, and demographic characteristics of the participants, including age and sex. Data related to peri‐implant diseases comprised the diagnostic criteria used and overall incidence at implant levels.

Information regarding soft tissue conditions around implants was also recorded, including the presence or absence of keratinized mucosa and, when available, the measured width of the keratinized mucosa. Additional variables included prosthetic characteristics (number of teeth replaced—single, multiple, or both ‐and type of retention, either screw‐retained or cement‐retained), implant characteristics (implant‐position platform, implant brand and surface type) and adjustment for confounding factors. In addition, patient‐related characteristics associated with peri‐implant disease risk, including supportive peri‐implant care, smoking status, and history of periodontitis, were extracted from the included studies when available. Information regarding the inclusion of multiple implants per patient and whether statistical methods accounting for clustering (e.g., GEE or multilevel models) were used was extracted from each study and summarized descriptively.

When essential data were incomplete or unclear, attempts were made to contact the corresponding authors for clarification or additional information. Any discrepancies between reviewers during the data collection process were resolved through discussion. In cases where consensus could not be achieved, a third and fourth reviewer (INRR and NN) were consulted to arbitrate the final decision.

### 2.7 Risk of Bias and Certainty of Evidence

Risk of bias was evaluated using the Joanna Briggs Institute (JBI) Critical Appraisal Checklist for Cohort Studies [17]. This appraisal was performed independently by two reviewers (MQP and TES). After completing the assessments, the reviewers compared their judgments and discussed any discrepancies. When consensus could not be reached, a third reviewer (CMP) was consulted to make the final decision.

The checklist comprises 11 items addressing key methodological aspects, including the comparability of exposed and non‐exposed groups and their recruitment from a common population, the accuracy and reliability of exposure and outcome measurements, confirmation that participants were outcome‐free at baseline, identification of potential confounding factors and strategies used to control them, adequacy of follow‐up duration, management and reporting of losses to follow‐up, and the suitability of the statistical analyses performed. An overall score was calculated for each study and used to determine its risk of bias. Based on the proportion of affirmative responses, studies were classified as having a low (≥ 70%), moderate (50%–69%), or high (≤ 49%) risk of bias, with higher scores reflecting better methodological quality and a lower risk of bias.

The certainty of evidence for each outcome was appraised using the Grading of Recommendations Assessment, Development and Evaluation (GRADE) framework [18] supported by the GRADEpro software (McMaster University, 2016). The overall certainty ratings were based on a structured evaluation of key domains, including methodological limitations across the included studies, variability and consistency of results, precision, and the degree of directness of the evidence.

### 2.8 Statistical Analysis and Data Synthesis

Initially, a descriptive synthesis of the included studies was performed. All quantitative analyses were conducted using Review Manager (RevMan) software (version 5.4.1). Separate meta‐analyses were carried out for peri‐implant mucositis and peri‐implantitis, considering implant‐level outcomes and different cutoff values for the width of keratinized mucosa. Subgroup analyses were conducted according to study design, that is, prospective and retrospective cohort studies. Between‐subgroup differences were evaluated to explore potential heterogeneity. When no statistically significant subgroup effect was observed, the overall pooled estimate was reported [19]. Random‐effects models were applied to account for anticipated clinical and methodological heterogeneity among the included studies. Considering that keratinized mucosa width is a site‐specific anatomical feature that may vary among implants within the same individual, analyses were performed at the implant level.

To explore the potential influence of follow‐up duration on the outcomes, subgroup analyses were performed according to follow‐up length (≤ 5 years vs. > 5 years). Studies were classified based on the reported mean or general follow‐up duration.

Furthermore, to account for the anticipated clinical heterogeneity regarding the bone level thresholds across the included studies, additional prespecified subgroup analyses were performed. The primary studies were stratified into three distinct diagnostic categories (incorporating marginal bone loss [MBL] and probing depth [PD] thresholds): (1) MBL > 0.5 mm; (2) MBL 1.5 to 2 mm; and (3) MBL ≥ 3 mm. In the latter two categories, the MBL thresholds were often combined with the requirement of deep peri‐implant pockets.

The results were expressed as pooled risk ratios (RRs) with corresponding 95% confidence intervals (CIs). Statistical heterogeneity was assessed using Cochran's *Q* test and quantified with the *I* ^2^ statistic.

## 3 Results

### 3.1 Selection of Eligible Studies

**FIGURE 1:**
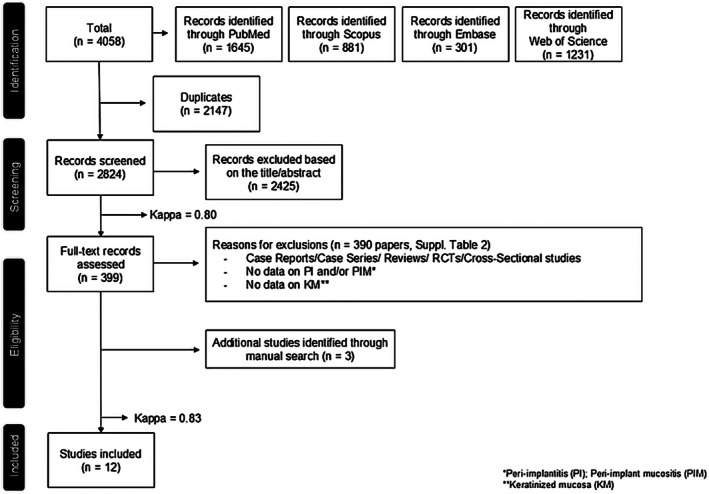
PRISMA flowchart of the study selection process.

The flowchart of the study selection process is presented in Figure 1. Initially, 4058 records were identified. After removal of 2147 duplicates, 2824 records remained for title and abstract screening. Full‐text assessment was performed for 399 publications, and three additional articles were identified through manual search. In total, 12 articles met the inclusion criteria. The excluded studies are listed in Table S2. Inter‐examiner agreement for the title and abstract screening, as assessed by the kappa coefficient, was 0.80, while agreement for the full‐text review was 0.83.

### 3.2 Characteristics of the Included Studies

Twelve prospective and retrospective cohort studies met the inclusion criteria comprising 938 patients and 2646 implants. As displayed in Table 1, studies were published between 2016 and 2025 and conducted in Brazil (*n* = 3) [20, 21, 22], Italy (*n* = 3) [23, 24, 25], Spain (*n* = 2) [26, 27], Iran (*n* = 1) [28], Turkey (*n* = 1) [29], Switzerland (*n* = 1) [30], and China (*n* = 1) [31]. Eight studies were conducted in university environments [20, 22, 24, 26, 27, 28, 29, 30, 31], one in a private clinic [25], one in a hospital [21], and one in both hospital and an academic setting [23]. The follow‐up periods ranged between one [22] to 20 years [25].

**TABLE 1:** Descriptive characteristics of the included studies.

References	Study design	Country	Clinical setting (private practice/hospital/university)	Follow‐up duration (years)	Implant functional time (mean, years)	Mean age	Sex distribution (Male/Female, n/%)	Number of patients (baseline/final)	Number of implants (baseline/final)
Alhakeem et al. [28]	Retrospective cohort	Iran	University	6–8 years	7.3 ± 1.4 years	39.0 ± 6.0 years	Male: 34 (38.64%) Female: 54 (61.36%)	88/88	186/186
Bozkurt et al. [29]	Retrospective cohort	Turkey	University	1–3.83 years	2.15 ± 0.73 years	NR	Male: 88 (52.1%) Female: 81 (47.9%)	169/169	645/645
Costa and Cota [20]	Prospective cohort	Brazil	University	11 years	~18 years	60.8 ± 10.5 years	Male: 23 (45.1%) Female: 28 (54.9%)	80/51	207/199
Felice et al. [23]	Retrospective cohort	Italy	Hospital and University	4.1 years	4.1 years SD was not reported	58.61 ± 9.60 years	Male: 39 (35.45%) Female: 71 (64.55%)	110/110	217/217
Fernandes et al. [21]	Retrospective cohort	Brazil	Hospital	6.2 years	6.20 ± 2.10 years	68.04 ± 9.07 years	Male: 38 (58.46%) Female: 27 (41.54%)	65/65	114/114
Mancini et al. [30]	Prospective cohort	Switzerland	University	10 years	10 years SD was not reported	63.4 ± 13.5 years	Male: 36 (48.6%) Female: 38 (51.4%)	74/74	148/148
Nícoli et al. [22]	Prospective cohort	Brazil	University	1 year	1 year SD was not reported	56.67 ± 8.44 years	Male: 22 (40.74%) Female: 32 (59.25%)	68/54	173/172
Lv et al. [31]	Retrospective cohort	China	University	6.4 years	6.4 years SD was not reported	53.3 ± 9.8 years	Male: 44 (50%) Female: 44 (50%)	88/88	182/182
Poli et al. [24]	Retrospective cohort	Italy	University	≥ 1 year	NR	60 ± 8.6 years	Male: 41 (39.8%) Female: 62 (60.2%)	103/103	421/421
Roccuzzo et al. [25]	Prospective cohort	Italy	Private practice	20 years	20 years SD was not reported	51.4 years	Male: 36 (56.25%) Female: 28 (43.75%)	128/64	128/64
Romandini et al. [5]	Prospective cohort	Spain	University	3.9 years	NR	62.8 years	Male: 28 (38.4%) Female: 45 (61.6%)	99/73	458/322
Ruiz‐Romero et al. [27]	Retrospective cohort	Spain	University	1.3–9.8 years	4.8 ± 2.0 years	58.7 years	Male: 36 (40.9%) Female: 52 (59.1%)	88/88	213/213

The number of participants per study ranged from 54 [22] to 169 [29], with mean patient ages varying from 39 [28] to 68 years [21]; while one study did not report data on patient age. The number of implants investigated per study ranged from 114 [21] to 645 [29].

With respect to keratinized mucosa, some studies provided data using a single cutoff value (either 0 or 2 mm). Data based on a cutoff of > 2 mm were available in 10 studies [20, 22, 23, 26, 27, 28, 29, 30, 31], while data for a cutoff of > 0 mm were available in six studies [21, 22, 24, 25, 26, 30].

Implant‐ and prosthesis‐related variables are detailed in Table 2. All included studies reported on implants supporting fixed restorations. Eight of the 12 studies exclusively evaluated fixed restorations (100%). Four studies also included removable prostheses, although fixed restorations represented the majority of all cases [26, 27, 29, 30]. Four studies [21, 22, 26, 29] reported a predominance of multiple‐unit prostheses, with FDPs representing the most frequent configuration, followed by full‐arch restorations in a smaller proportion of cases. In contrast, the remaining studies included a mixed distribution of single and multiple‐unit restorations [27, 30, 31].

**TABLE 2:** Implant and prosthetic‐related characteristics reported in the included studies.

References	Number of patients (baseline/final)	Number of implants (baseline/final)	Keratinized mucosa threshold used in the study (mm)	Implant platform position (crestal/supracrestal, at the tissue level) number/%	Implant brand	Implant surface characteristics	Prosthesis configuration (single and/or multiple) number/%	Prosthesis type (removable and/or fixed) number/%	Retention method (cement‐retained and/or screw‐retained) number/%
Alhakeem et al. [28]	88/88	186/186	≥ 2 mm	Bone level: 160/86.02% Tissue Level: 26/13.98%	Straumann, Dentium	Modified	NR	Fixed: 186/100%	NR
Bozkurt et al. [29]	169/169	645/645	≥ 2 mm	NR	Straumann, Astra Tech, Nobel, Zinedent, MIS	Modified	Single: 104/16.1% Bridge: 500/77.5% Full arch: 41/6.4%	Fixed: 604/93.6% Removable: 41/6.4%	NR
Costa and Cota [20]	80/51	207/199	> 0 mm	NR	NR	Modified	NR	Fixed: 207/100%	NR
Felice et al. [23]	110/110	217/217	≥ 2 mm	NR	NR	Modified	Single and multiple (short‐span partial)	Fixed: 217/100%	NR
Fernandes et al. [21]	65/65	114/114	> 0 mm	NR	Nobel Biocare (Mk III and Replace Select), Conexão Sistema de Prótese (Master Easy), Biomet 3i: 1 implant, Dentsply Sirona Ankylos: 1 implant	Modified	Single: 15/13.51% Multiple: 93/86.49%	Fixed:111/100%	Cement‐retained: 45/40.54% Screw‐retained: 66/59.46%
Mancini et al. [30]	74/74	148/148	≥ 2 mm and > 0 mm	Bone level: 101/68.2% Tissue level: 47/31.8%	Astra Tech Osseospeed, Straumann Bone Level, Tissue Level, Branemark MKIII/MKIV	Modified	Single: 63/42.6% Multiple: 77/52%	Fixed: 140/94.6% Removable: 8/5.4%	Cement‐retained: 20/13.5% Screw‐retained: 128/86.5%
Nícoli et al. [22]	54/53	173/172	≥ 2 mm	NR	Implacil de Bortoli, Conexão Sistemas de Próteses, Neodent and Bionovation	Modified	Single: 51/29.5% Multiple: 122/70.5%	Fixed: 173/100%	Cement‐retained: 13/7.2% Screw‐retained: 165/95.4%
Lv et al. [31]	88/88	182/182	≥ 2 mm	Bone level: 96/52.7% Tissue level: 86/47.3%	Straumann, Bicon	Modified	Single and multiple Number NR	Fixed: 182/100%	NR
Poli et al. [24]	103/103	421/421	> 0 mm	NR	NR	Modified	Single and multiple Number NR	Fixed Number NR	NR
Roccuzzo et al. [25]	128/64	128/64	> 0 mm	Tissue level: 128/100%	Straumann Group AG, Basel, Switzerland (SLA surface)	Modified	Single: 128/100%	Fixed: 64/100%	Cement‐retained: 64/100%
Romandini et al. [5]	73/73	322/298	≥ 2 mm and > 0 mm	Bone level	Straumann, Nobel Biocare, AstraTech, Other	Modified	Single: 103/32% Multiple: 219/68.0%	Fixed: 314/97.5% Removable: 8/2.5%	Cement‐retained: 163/50.6% Screw‐retained: 151/46.9% Overdenture (bar/locator): 8/2.5%
Ruiz‐Romero et al. [27]	88/88	213/213	≥ 2 mm	Bone level: 213/100%	Avinent (Avinent Dental System, Santpedor, Spain)	Modified	Single: 106/49.8% Multiple: 82/38.5% Multiple (overdenture): 25/11.7%	Fixed: 188/88.3% Removable: 25/11.7%	Screw‐retained

Retention methods were reported in six studies. Four studies included both cemented and screw‐retained prostheses [21, 22, 26, 30]. Among these, two studies reported a predominance of screw‐retained restorations [22, 30], while two studies reported a more balanced distribution between cemented and screw‐retained restorations [21, 26]. One study used exclusively cement‐retained restorations [25], and one study used exclusively screw‐retained restorations [27]. Five studies did not report on the retention method [20, 23, 24, 28, 29, 31].

Table 3 summarizes the patient‐related characteristics reported in the included studies, including supportive peri‐implant care, smoking status, and history of periodontitis. Reporting of these variables varied across studies, with several cohorts not providing complete information for all factors.

**TABLE 3:** Patient‐related characteristics reported in the included studies.

References	Supportive peri‐implant care (SPC)	Smoking (number/%)	History of periodontitis (number/%)
Alhakeem et al. [28]	Regular and irregular recall intervals	1 (1.1%)	47 (53.4%)
Bozkurt et al. [29]	Not evaluated	0 (0%)	Not reported
Costa and Cota [20]	Regular compliers (100% compliant, max interval 9 months) and irregular compliers (missed visits, max interval 18 months)	0 (0%)	28 (54.9%)
Felice et al. [23]	Not evaluated	16 (14.5%)	Not reported
Fernandes et al. [21]	Not evaluated	0 (0%)	Not reported
Mancini et al. [30]	Compliers (≥ 2 visits/year) and non‐compliers (< 2 visits/year)	Not reported	Not reported
Nícoli et al. [22]	Not evaluated	4 (7.1%)	Not reported
Lv et al. [31]	Only assessed patient's self‐performed proximal cleaning habits (fair vs. poor), but not professional SPC.	12 (13.6%)	88 (100%)
Poli et al. [24]	Professional oral hygiene recalls: < 6 months vs. > 6 months	Only implant‐level data: 77 of 421 implants (18.3%)	Not reported
Roccuzzo et al. [25]	Adherence to SPC (Yes/No), defined as full compliance with the individualized program	6 (14.3%)	40 (62.5%)
Romandini et al. [5]	Number of maintenances; regular maintenance (≥ 1 visit/year)	14 (19.2%)	63 (86.3%)
Ruiz‐Romero et al. [27]	Excluded all patients attending ≥ 2 SPC appointments/year. Entire sample consisted of erratic/non‐compliers.	19 (21.6%)	37 (42.0%)

The diagnostic criteria adopted for peri‐implant mucositis and peri‐implantitis, along with the corresponding follow‐up periods, are outlined in Table 4. All 12 included studies defined peri‐implant mucositis based on the presence of bleeding on probing (BOP) and/or suppuration (SUP) measured at six points per implant, in the absence of marginal bone loss beyond initial physiological remodeling. For the diagnosis of peri‐implantitis, all studies required the presence of inflammatory signs (BOP and/or SUP) combined with marginal bone loss (MBL); however, the radiographic thresholds used to define bone loss varied across studies, although all diagnoses were based on longitudinal radiographic evaluation.

**TABLE 4:** Definitions of peri‐implant mucositis and peri‐implantitis.

References	Definition of peri‐implant mucositis	Definition of peri‐implantitis
Alhakeem et al. [28]	BOP/SUP+	BOP/SUP+ MBL ≥ 3 mm (longitudinal assessment—previous radiograph) PD ≥ 4 mm
Bozkurt et al. [29]	BOP/SUP+	BOP/SUP+ MBL > 0.5 mm (longitudinal assessment—previous radiograph)
Costa and Cota [20]	BOP/SUP+	BOP/SUP+ MBL ≥ 3 mm (longitudinal assessment—previous radiograph) PD ≥ 6 mm
Felice et al. [23]	BOP/SUP+	BOP/SUP+ MBL > 0.5 mm (longitudinal assessment—previous radiograph) Increase in PD compared to previous examinations
Fernandes et al. [21]	BOP/SUP+	BOP/SUP+ MBL > 1.5 mm (longitudinal assessment—previous radiograph)
Mancini et al. [30]	BOP/SUP+	BOP/SUP+ MBL > 0.5 mm (longitudinal assessment—previous radiograph) Increase in PD compared to previous examinations
Nícoli et al. [22]	BOP/SUP+	BOP/SUP+ MBL ≥ 2 mm (longitudinal assessment—previous radiograph) PD ≥ 5 mm
Lv et al. [31]	BOP/SUP+	BOP/SUP+ MBL > 0.5 mm (longitudinal assessment—previous radiograph)
Poli et al. [24]	BOP/SUP+	BOP/SUP+ MBL: ≥ 2 mm (longitudinal assessment—previous radiograph) PD: ≥ 4 mm
Roccuzzo et al. [25]	BOP/SUP+	BOP/SUP+ MBL > 0.5 mm (longitudinal assessment—previous radiograph)
Romandini et al. [5]	BOP/SUP+	BOP/SUP+ MBL > 0.5 mm (longitudinal assessment—previous radiograph) (provided by the author)
Ruiz‐Romero et al. [27]	BOP/SUP+	BOP/SUP+ MBL > 0.5 mm (longitudinal assessment—previous radiograph) In the absence of previous examinations: BOP/SUP+ MBL ≥ 3 mm PD ≥ 6 mm

Table 5 summarizes additional methodological information from the included studies, particularly regarding the assessment of keratinized mucosa, the analytical management of multiple implants per patient, the use of adjusted multivariable models, control for confounding, follow‐up characteristics, and the handling of incomplete or missing data.

**TABLE 5:** Additional methodological information.

References	Study design	Keratinized mucosa threshold used in the study (mm)	Site of KM assessment	Adjustment for clustered data (multiple implants)	Multivariable model	Covariates included in the models (adjustments)	Adjusted effect estimate for KM reported in the final multivariable model	Adjustment for confounding factors (Yes/No)	Follow‐up duration (years)	Follow‐up completeness (attrition rate)	Handling of missing data
Alhakeem et al. [28]	Retrospective cohort	≥ 2 mm	Periodontal probe; Buccal aspect	Yes, generalized estimating equations	Yes, generalized estimating equations	History of periodontitis, age, sex/gender, interdental contact, smoking, brushing frequency, recall interval, plaque score, bleeding score, splinted prosthesis, implant type, mucosal thickness, bone grafting	Yes (Adjusted OR = 14.94 for lack of KM)	Yes	6–8 years	48.2% non‐attenders (retrospective design, retrospective recall exam)	Exclusion (listwise deletion); no imputation used
Bozkurt et al. [29]	Retrospective cohort	≥ 2 mm	Periodontal probe; Mid‐buccal site	Implant‐level analysis (no clustering adjustment reported)	No	None	No	No	1–3.83 years	Unclear (retrospective design, retrospective chart review)	Exclusion of incomplete records
Costa and Cota [20]	Prospective cohort	> 0 mm	Periodontal probe; Mid‐buccal and mid‐lingual site^a^	Implant‐level analysis (no clustering adjustment reported)	Yes, logistic regression	Age, sex, plaque index, peri‐implant mucositis, periodontitis, time of prosthesis installation, missing teeth, compliance	No (KM dropped from final model due to non‐significance)	Yes	11 years	36.2% loss to follow‐up	Exclusion of non‐attenders; no imputation used
Felice et al. [23]	Retrospective cohort	≥ 2 mm	Periodontal probe; Mid‐buccal site	Yes, multilevel regression	Yes, multilevel logistic	Smoking, arch, implant length, implant diameter.	Yes. Adjusted OR = 2.76 (95% CI 0.42–17.99; *p* = 0.29) for implant failure due to peri‐implantitis. Adjusted OR = 3.03 (95% CI 0.68–13.46; *p* = 0.14) for overall complications. Overall complications included one case of peri‐implant mucositis and five cases of peri‐implantitis.	Yes	4.1 years	Unclear (retrospective design, retrospective chart review)	Exclusion of incomplete records
Fernandes et al. [21]	Retrospective cohort	> 0 mm	Periodontal probe; Mid‐buccal site	Implant‐level analysis (no clustering adjustment reported)	No	None	No	No	6.2 years	Unclear (retrospective design, retrospective chart review)	Exclusion of incomplete records
Mancini et al. [30]	Prospective cohort	≥ 2 mm and > 0 mm	Periodontal probe; Mid‐buccal site	Yes, generalized estimating equations	Yes, generalized estimating equations	Compliance, implant system (one‐piece vs. two‐piece), gender, implant position, age.	Yes (Adjusted OR = 0.65 for presence of KM/non‐significant for peri‐implantitis).	Yes	10 years	Unclear	Complete‐case analysis (Exclusion); no imputation used
Nícoli et al. [22]	Prospective cohort	≥ 2 mm	Periodontal probe; Mid‐buccal site	Implant‐level analysis (no clustering adjustment reported)	Yes, multivariate linear regression	Gender, type of prosthesis, width of keratinized mucosa.	No (No peri‐implantitis cases observed).	Yes	1 year	20.5% loss to follow‐up	Exclusion of participants with missing data
Lv et al. [31]	Retrospective cohort	≥ 2 mm	Not specified	Implant‐level analysis (no clustering adjustment reported)	Yes, logistic regression	Age, gender, proximal cleaning, implant type, position, periodontal disease control, peri‐implant plaque index.	Yes (Adjusted OR = 19.20 for KTW < 2 mm).	Yes	6.4 years	Unclear (retrospective design, retrospective chart review)	Exclusion of incomplete records
Poli et al. [24]	Retrospective cohort	> 0 mm	Periodontal probe and visual assessment; Mid‐buccal site and mid‐oral site	Implant‐level analysis (no clustering adjustment reported)	Yes, logistic regression	Age, gender, smoking, oral hygiene recalls, biotype, membrane, bone graft, plaque, soft tissue recession.	Yes (Adjusted OR = 3.99 for absence of KM).	Yes	≥ 1 year	Unclear (retrospective design, retrospective chart review)	Exclusion of incomplete records
Roccuzzo et al. [25]	Prospective cohort	> 0 mm	Visual assessment; Mid‐buccal site	Not applicable (1 implant per patient)	Yes, binary logistic	Group, sex, age, smoking, supportive periodontal/peri‐implant care, moderate periodontally compromised patients	Yes (Adjusted OR = 19.1 for AM group vs. KT/Grafted group)	Yes	20 years	50.0% loss to follow‐up	Exclusion of non‐attenders; no imputation used
Romandini et al. [5]	Prospective cohort	≥ 2 mm and > 0 mm	Periodontal probe; Mid‐buccal site^a^	Yes, multilevel mixed‐effects	Yes, multilevel logistic	Periodontitis severity, smoking, sleep duration, implant location, restoration type, margin location, plaque, bone loss/age ratio, among 80+ variables tested.	No (KM dropped from final model due to non‐significance).	Yes	3.9 years	26.2% loss to follow‐up	Exclusion of non‐attenders and missing radiographs
Ruiz‐Romero et al. [27]	Retrospective cohort	≥ 2 mm	Periodontal probe; Mid‐buccal, mesial, and distal line angles	Yes, multilevel logistic regression analyses	Yes, generalized estimating equations	Diabetes mellitus, prosthesis emergence angle, baseline marginal bone loss.	Yes (Adjusted OR = 5.26 for KM < 2 mm).	Yes	1.3–9.8 years	24.1% excluded (retrospective design, inadequate follow‐up/records)	Exclusion of inadequate follow‐ups; no imputation used

^a^ Information obtained from the authors upon request.

### 3.3 Meta‐Analysis

#### 3.3.1 Peri‐Implant Mucositis Risk Comparing Implants With (> 0 mm) and Without Keratinized Mucosa (0 mm)

Five studies were included in the implant‐level meta‐analysis comparing the risk of peri‐implant mucositis between implants with keratinized mucosa present (> 0 mm) and those without keratinized mucosa (0 mm). Five cohort studies [22, 24, 25, 26, 30] contributed data to this analysis.

Subgroup analyses were performed according to study design. In the prospective cohort subgroup (*n* = 4 studies), no statistically significant difference in the risk of peri‐implant mucositis was observed between implants with and without keratinized mucosa (RR: 0.91; 95% CI: 0.60–1.39), with substantial heterogeneity among studies (*I* ^2^ = 73%). Similarly, one retrospective cohort study showed no significant difference between the groups (RR: 0.90; 95% CI: 0.70–1.16).

**FIGURE 2:**
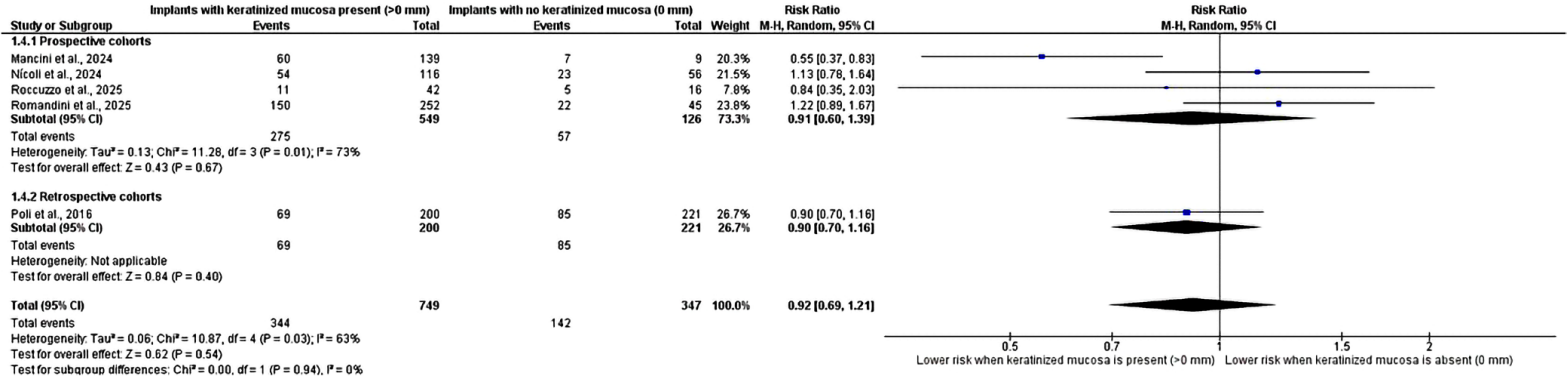
Forest plot of the meta‐analysis comparing the risk of peri‐implant mucositis between implants with keratinized mucosa present (> 0 mm) and implants without keratinized mucosa (0 mm).

When all studies were pooled, the overall meta‐analysis demonstrated no significant association between the presence of keratinized mucosa and the risk of peri‐implant mucositis at the implant level (RR: 0.92; 95% CI: 0.69–1.21; *p* = 0.54), with moderate heterogeneity (*I* ^2^ = 63%). No significant differences were observed between subgroups based on study design (*p* = 0.94). The results of this analysis are illustrated in Figure 2.

#### 3.3.2 Peri‐Implant Mucositis Risk Comparing Implants With Keratinized Mucosa ≥ 2 mm and < 2 mm

Seven studies were included in the implant‐level meta‐analysis comparing the risk of peri‐implant mucositis between implants with a keratinized mucosa width ≥ 2 mm and those with < 2 mm. Seven cohort studies [20, 22, 23, 26, 27, 29, 30] contributed data to this analysis.

In the prospective cohort subgroup (*n* = 4 studies), no statistically significant difference in the risk of peri‐implant mucositis was observed between implants with ≥ 2 mm and < 2 mm of keratinized mucosa (RR: 0.60; 95% CI: 0.27–1.32), with considerable heterogeneity among studies (*I* ^2^ = 96%). Similarly, the retrospective cohort subgroup (*n* = 3 studies) showed no significant difference between the groups (RR: 0.98; 95% CI: 0.74–1.28), with no observed heterogeneity (*I* ^2^ = 0%).

**FIGURE 3:**
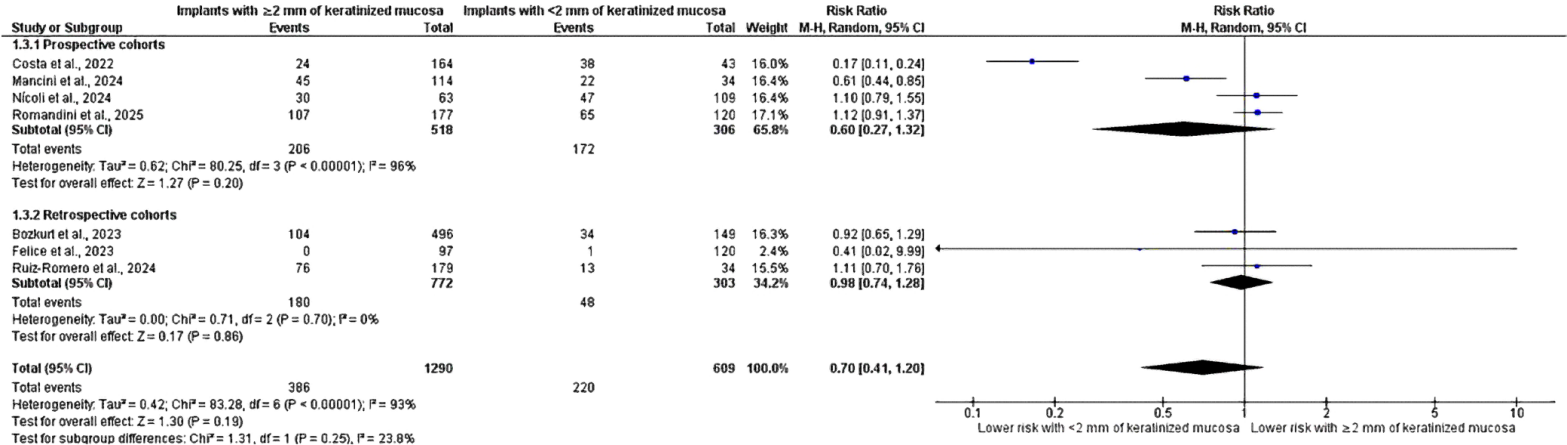
Forest plot of the meta‐analysis comparing the risk of peri‐implant mucositis between implants with keratinized mucosa ≥ 2 mm and implants with keratinized mucosa < 2 mm.

When all studies were pooled, the overall meta‐analysis demonstrated no significant association between the width of keratinized mucosa and the risk of peri‐implant mucositis at the implant level (RR: 0.70; 95% CI: 0.41–1.20; *p* = 0.19), although substantial heterogeneity was observed (*I* ^2^ = 93%). No statistically significant differences were detected between subgroups based on study design (*p* = 0.25). The results of this analysis are presented in Figure 3.

#### 3.3.3 Peri‐Implantitis Risk Comparing Implants With (> 0 mm) and Without Keratinized Mucosa (0 mm)

Six cohort studies [21, 22, 24, 25, 26, 30] were included in the implant‐level meta‐analysis comparing the risk of peri‐implantitis between implants with keratinized mucosa present (> 0 mm) and those without keratinized mucosa (0 mm). Six cohort studies contributed data to this analysis.

In the prospective cohort subgroup (*n* = 4 studies), no statistically significant difference in the risk of peri‐implantitis was observed between implants with and without keratinized mucosa (RR: 0.64; 95% CI: 0.31–1.31), with low heterogeneity (*I* ^2^ = 38%). In the retrospective cohort subgroup (*n* = 2 studies), no statistically significant difference in peri‐implantitis risk was observed between implants with and without keratinized mucosa (RR: 0.46; 95% CI: 0.21–1.03), with no observed heterogeneity (*I* ^2^ = 0%).

**FIGURE 4:**
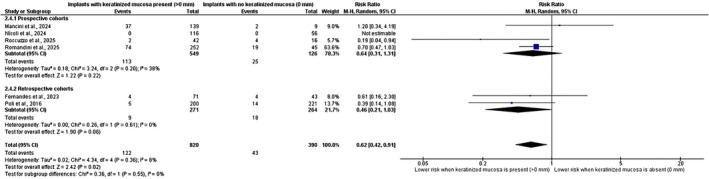
Forest plot of the meta‐analysis comparing the risk of peri‐implantitis between implants with keratinized mucosa present (> 0 mm) and implants without keratinized mucosa (0 mm).

When all studies were pooled, the overall meta‐analysis demonstrated a significantly lower risk of peri‐implantitis for implants with keratinized mucosa compared with those without keratinized mucosa at the implant level (RR: 0.62; 95% CI: 0.42–0.91; *p* = 0.02), with low heterogeneity among studies (*I* ^2^ = 8%). No significant differences were observed between subgroups based on study design (*p* = 0.55). The results of this analysis are illustrated in Figure 4.

#### 3.3.4 Peri‐Implantitis Risk Comparing Implants With Keratinized Mucosa ≥ 2 mm and With < 2 mm of Keratinized Mucosa

Nine studies [20, 22, 23, 26, 27, 28, 29, 30, 31] were included in the implant‐level meta‐analysis comparing the risk of peri‐implantitis between implants presenting a width of keratinized mucosa ≥ 2 mm compared to < 2 mm.

In the prospective cohort subgroup (*n* = 4 studies), no statistically significant difference in the risk of peri‐implantitis was observed between implants with ≥ 2 mm and < 2 mm of keratinized mucosa (RR: 0.64; 95% CI: 0.34–1.19), although substantial heterogeneity was detected (*I* ^2^ = 81%). The retrospective cohort subgroup (*n* = 5 studies) demonstrated a significantly lower risk of peri‐implantitis for implants with ≥ 2 mm of keratinized mucosa compared with those with < 2 mm (RR: 0.49; 95% CI: 0.26–0.94; *p* = 0.03), with moderate heterogeneity (*I* ^2^ = 62%).

**FIGURE 5:**
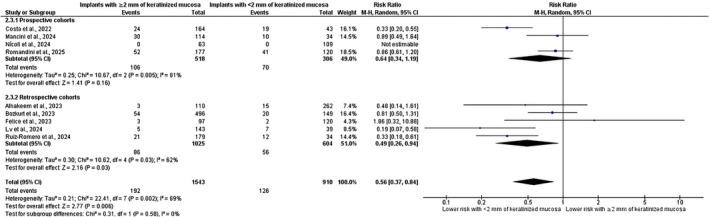
Forest plot of the meta‐analysis comparing the risk of peri‐implantitis between implants with ≥ 2 mm of keratinized mucosa and implants with < 2 mm of keratinized mucosa.

When all studies were pooled, the overall meta‐analysis showed a significantly reduced risk of peri‐implantitis associated with a width of keratinized mucosa of ≥ 2 mm at the implant level (RR: 0.56; 95% CI: 0.37–0.84; *p* = 0.006), despite moderate heterogeneity across studies (*I* ^2^ = 69%). No statistically significant differences were observed between subgroups based on study design (*p* = 0.58). The results of this analysis are presented in Figure 5.

#### 3.3.5 Subgroup Analyses by Follow‐Up Duration (≤ 5 Years vs. > 5 Years)

Subgroup analyses according to follow‐up duration were performed to explore whether follow‐up length influenced the pooled estimates (Figures S1–S4). Overall, the pattern of associations was broadly similar across follow‐up subgroups.

For peri‐implant mucositis, no significant associations were observed in either comparison. Studies with longer follow‐up (> 5 years) showed larger (more protective) effect estimates associated with keratinized mucosa compared with studies with ≤ 5 years, although these differences were not statistically significant.

For peri‐implantitis, a protective pattern associated with keratinized mucosa was observed across both follow‐up periods. In both comparisons (> 0 mm vs. 0 mm and ≥ 2 mm vs. < 2 mm), the magnitude of the protective association tended to be greater in studies with longer follow‐up, suggesting a possible time‐dependent effect, although subgroup differences were not statistically significant.

#### 3.3.6 Subgroup Analyses by Peri‐Implantitis Diagnostic Thresholds (MBL and PD)

Subgroup analyses according to the diagnostic thresholds used to define peri‐implantitis were performed for the comparison between the presence (> 0 mm) and absence (0 mm) of keratinized mucosa (Figure S5). The direction of the association remained consistent across the two definitions, with no significant differences between subgroups (*p* = 0.55).

In the comparison between ≥ 2 and < 2 mm of keratinized mucosa, implants with ≥ 2 mm showed a significantly lower risk of peri‐implantitis (RR = 0.56, 95% CI 0.37–0.84). Larger (more protective) effect estimates were observed in studies applying more stringent diagnostic thresholds; however, the differences between subgroups did not reach statistical significance (*p* = 0.07) (Figure S6).

### 3.4 Risk of Bias

The quality assessment of results from the included studies is summarized in Table 6, with details of the analysis provided in Table S3. Risk of bias assessment using The JBI Critical Appraisal Tool for Cohort Studies indicated a low risk of bias in nine studies (75%) [20, 22, 23, 24, 25, 26, 27, 28, 30], two studies (16.7%) with a moderate risk of bias [29, 31], and one study (8.3%) with a high risk of bias [21].

**TABLE 6:** Risk of bias assessment according to Joanna Briggs for cohort studies.

Studies	Q1	Q2	Q3	Q4	Q5	Q6	Q7	Q8	Q9	Q10	Q11	Total%	RoB
Alhakeem et al. [28]	U	Y	Y	Y	Y	N	Y	Y	Y	N	Y	72.73	Low
Bozkurt et al. [29]	U	Y	Y	Y	N	Y	Y	Y	U	N	N	54.55	Moderate
Costa and Cota [20]	Y	Y	Y	Y	Y	Y	Y	Y	N	N	Y	81.82	Low
Felice et al. [23]	Y	Y	Y	Y	Y	Y	Y	Y	U	U	Y	81.82	Low
Fernandes et al. [21]	U	Y	Y	Y	N	N	Y	Y	U	N	N	45.44	High
Mancini et al. [30]	U	Y	Y	Y	Y	Y	Y	Y	U	N	Y	72.73	Low
Nícoli et al. [22]	U	Y	Y	Y	Y	Y	Y	Y	Y	N	Y	81.82	Low
Lv et al. [31]	U	Y	U	Y	Y	Y	Y	Y	U	U	Y	63.64	Moderate
Poli et al. [24]	Y	Y	Y	Y	Y	U	Y	Y	N	N	Y	72.73	Low
Roccuzzo et al. [25]	U	Y	Y	Y	U	Y	Y	Y	Y	N	Y	72.73	Low
Romandini et al. [5]	U	Y	N	Y	Y	Y	Y	Y	Y	NA	Y	72.73	Low
Ruiz‐Romero et al. [27]	U	Y	Y	Y	Y	N	Y	Y	Y	N	Y	72.73	Low

*Note:* Q1: Were the two groups similar and recruited from the same population?; Q2: Were the exposures measured similarly to assign people to both exposed and unexposed groups?; Q3: Was the exposure measured in a valid and reliable way?; Q4: Were confounding factors identified?; Q5: Were strategies to deal with confounding factors stated?; Q6: Were the groups/participants free of the outcome at the start of the study (or at the moment of exposure)?; Q7: Were the outcomes measured in a valid and reliable way?; Q8: Was the follow up time reported and sufficient to be long enough for outcomes to occur?; Q9: Was follow up complete, and if not, were the reasons to loss to follow up described and explored?; Q10: Were strategies to address incomplete follow up utilized?; and Q11: Was appropriate statistical analysis used?.

Abbreviations: N, no; NA, not applicable; U, unclear; Y, yes.

### 3.5 Certainty of Evidence

The certainty of evidence according to the GRADE approach is presented in Table S4. For peri‐implant mucositis, the certainty of evidence was rated as very low for both comparisons (> 0 mm vs. 0 mm and ≥ 2 mm vs. < 2 mm), due to the observational design of the included studies and serious to very serious inconsistency and imprecision, as reflected by substantial heterogeneity and wide confidence intervals crossing the null effect.

For peri‐implantitis, the certainty of evidence was rated as low for the comparison between ≥ 2 mm and < 2 mm, mainly due to the observational nature of the evidence. In contrast, the certainty of evidence was rated as very low for the comparison between implants with keratinized mucosa present (> 0 mm) and absent (0 mm), due to the observational nature of the evidence in terms of inconsistency and imprecision across studies.

## 4 Discussion

### 4.1 Summary of Main Findings

The present systematic review and meta‐analysis evaluated the incidence of peri‐implant diseases in relation to the presence and width of keratinized mucosa at the implant level, exclusively based on longitudinal cohort studies. Albeit, a plethora of systematic reviews reported on this topic, the present SR is the first to separately assess two keratinized mucosa thresholds (presence vs. absence and ≥ 2 mm vs. < 2 mm), thereby allowing a temporal and more refined evaluation of peri‐implant disease development.

Overall, the findings indicate that keratinized mucosa is not associated with the risk of peri‐implant mucositis, whereas a significant association was consistently observed for peri‐implantitis across both keratinized mucosa thresholds evaluated.

For peri‐implant mucositis, no significant association was identified either when comparing implants with and without keratinized mucosa (> 0 mm vs. 0 mm) or when different cutoff values for keratinized mucosa width (≥ 2 mm vs. < 2 mm) were applied. These results were consistent across study designs and were maintained in sensitivity analyses, suggesting that the presence or width of keratinized mucosa alone does not appear to be a determining factor for the development of peri‐implant mucositis.

In contrast, peri‐implantitis was significantly less frequent in implants surrounded by keratinized mucosa. A significantly lower risk of peri‐implantitis was observed both when keratinized mucosa was present (> 0 mm) compared with its absence and when a keratinized mucosa width of at least 2 mm was compared with narrower bands. The magnitude of the association was more pronounced for the ≥ 2 mm threshold, and this finding remained statistically significant in the overall analyses and in sensitivity analyses, supporting the consistency of this association.

### 4.2 Comparison With Previous Systematic Reviews

The relationship between keratinized mucosa and peri‐implant health has been explored in primary studies for several decades, often yielding divergent results. Part of this inconsistency may be explained by differences in the outcomes assessed and in the methodological characteristics of the studies included in previous systematic reviews. Most earlier reviews focused on isolated clinical parameters, such as bleeding on probing, plaque indices, or marginal bone levels, rather than on the occurrence of peri‐implant diseases as diagnostic entities and relied predominantly on non‐longitudinal data. For this reason, the analytical approaches adopted in previous systematic reviews make direct comparison with the present findings challenging.

A relatively consistent finding across previous reviews is the role of keratinized mucosa in plaque control and soft tissue health. Reviews have generally reported that implants surrounded by a reduced (< 2 mm) or an absent keratinized mucosa tend to exhibit higher plaque accumulation, increased bleeding indices, and greater signs of soft tissue inflammation [11, 32, 33, 34]. These findings support the concept that an adequate band of keratinized mucosa facilitates effective oral hygiene and contributes to peri‐implant soft tissue stability.

A point of stronger convergence among recent reviews relates to patient‐reported outcomes. Implants lacking an adequate band of keratinized mucosa have been consistently associated with increased discomfort or pain during tooth brushing [11, 12]. This discomfort, attributed to the mobility and sensitivity of non‐keratinized alveolar mucosa, may negatively affect oral hygiene practices and indirectly contribute to the progression of peri‐implant inflammatory conditions [8, 35]. Similarly, an association between the absence of keratinized mucosa and increased mucosal recession has been reported [11, 34].

In contrast, evidence regarding the influence of keratinized mucosa on supporting structures has been more heterogeneous. While some reviews have suggested an association between adequate keratinized mucosa and reduced marginal bone loss or improved clinical attachment levels [32, 34], others have failed to demonstrate significant differences in probing depth or bone loss [12, 33]. It may be speculated that focusing on individual structural measurements, without concomitant assessment of inflammatory signs, limits the ability to distinguish between clinically relevant disease processes and isolated findings. Consequently, evaluating bone‐related parameters outside a diagnostic framework that integrates both soft tissue inflammation and bone loss may contribute to the heterogeneous findings.

One previous systematic review addressed peri‐implant diseases as primary outcomes but was unable to perform a quantitative synthesis due to the limited availability of longitudinal data. The evidence was derived mainly from cross‐sectional studies, restricting the analysis to prevalence estimates and limiting causal inference [11].

Another systematic review with meta‐analysis specifically investigated the association between keratinized mucosa and peri‐implantitis prevalence. The quantitative synthesis was ultimately based exclusively on cross‐sectional studies, as only a very limited number of longitudinal investigations were available. Consequently, the meta‐analysis was restricted to prevalence estimates, which do not allow temporal or causal inferences. In addition, variability was observed in the cutoff values used to define keratinized mucosa across the included studies. While the pooled analysis suggested a higher prevalence of peri‐implantitis in implants lacking adequate keratinized mucosa, the reliance on cross‐sectional data and heterogeneous case definitions highlights important methodological constraints that should be considered when interpreting these findings [36].

Overall, heterogeneity in study design, outcome definitions, and analytical approaches across previous systematic reviews limits direct comparison with the present findings. Differences in study design, outcome metrics, and disease classification constrain interpretability. Therefore, rather than directly contrasting effect estimates, the present review should be interpreted as complementing the existing literature by addressing these methodological gaps through an incidence‐based approach.

### 4.3 Clinical Interpretation of the Findings

The observation that a significant association with peri‐implantitis was detected using both keratinized mucosa thresholds (> 0 mm and ≥ 2 mm) suggests that different aspects of keratinized mucosa may be relevant for peri‐implant tissue stability. The presence of any keratinized mucosa (> 0 mm) was associated with a reduction in peri‐implantitis risk corresponding to an approximate 38% relative risk reduction, highlighting the biological relevance of avoiding complete absence of keratinized tissue. In addition, the stronger association observed for the ≥ 2 mm threshold, corresponding to an approximate 44% relative risk reduction, suggests that wider bands of keratinized mucosa may confer additional protection, likely by facilitating plaque control [11, 32, 33, 34], reducing tissue trauma during oral hygiene [12, 35], and improving patient comfort, particularly in situations of suboptimal hygiene or irregular maintenance [37]. These findings suggest a potential dose–response relationship, in which wider bands of keratinized mucosa may offer incremental benefits, while also underscoring the biological importance of the presence of any keratinized tissue per se.

From a methodological and clinical perspective, each cutoff presents advantages and limitations. The ≥ 2 mm threshold has been widely adopted as a clinical reference point and may serve as a practical guide when considering soft tissue augmentation [9, 37]; however, it is subject to measurement imprecision and has been criticized as an arbitrary value extrapolated from periodontal literature [30]. In contrast, the dichotomous classification based on presence versus absence (> 0 mm vs. 0 mm) reduces measurement bias and highlights the high‐risk scenario represented by the complete lack of keratinized mucosa, although it may overlook situations in which very narrow bands are insufficient to ensure long‐term tissue stability [30]. In light of these considerations, our findings support the interpretation that both the presence and the width of keratinized mucosa are clinically relevant, reflecting complementary rather than competing perspectives. Long‐term data suggest that the complete absence of keratinized mucosa identifies a high‐risk scenario for peri‐implant disease [30], whereas wider bands, particularly around 2 mm, are associated with a higher likelihood of tissue adhesion and greater relative reductions in the risk of developing peri‐implantitis [37].

Beyond the quantitative assessment of keratinized mucosa width, growing evidence suggests that the functional characteristics of the peri‐implant soft tissue, particularly its degree of adhesion, may be more relevant than the absolute number of millimeters [30, 38]. From a biological and clinical perspective, keratinized mucosa appears to exert a protective role primarily when it is firmly attached to the underlying bone or implant surface, providing mechanical stability and resistance to displacement [30, 37, 39]. Narrow bands of keratinized mucosa, although visually present, are more likely to be mobile and behave functionally similar to alveolar mucosa, thereby offering limited protection against mechanical stress, plaque accumulation, and inflammation [30, 39, 40].

Recent conceptual frameworks have further emphasized the importance of tissue attachment in peri‐implant health [38, 41]. When the mucogingival junction is positioned apical to the crestal bone, the peri‐implant mucosa is more likely to be inserted and stable, whereas keratinized tissue located coronal to the bone crest may remain non‐adherent and susceptible to displacement under muscular tension [38, 39]. Rather than representing a biologically validated cutoff, the 2 mm threshold should be interpreted as a pragmatic classifier used in the literature to approximate the presence of an adequate band of adherent, non‐mobile mucosa [30, 37].

In front of this background, the lack of an observed association between keratinized mucosa and peri‐implant mucositis in the present study warrants further consideration from both anatomical and diagnostic perspectives. Unlike natural teeth, peri‐implant tissues lack periodontal ligament fibers inserting perpendicularly into the root surface; instead, collagen fibers are oriented parallel or circumferentially to the implant axis, and epithelial attachment relies on relatively weak hemidesmosomal junctions [39, 42, 43]. As a result, the peri‐implant soft tissue seal is more fragile and offers less resistance to probing, making bleeding more likely to occur due to mechanical trauma even in the absence of clinically relevant inflammation [39, 42, 43]. Consequently, distinguishing between true plaque‐induced mucosal inflammation and trauma‐induced bleeding becomes particularly challenging.

In this context, the diagnostic criteria used to define peri‐implant mucositis become particularly relevant. The studies included in this review consistently applied a threshold of at least one bleeding point on probing (BOP ≥ 1), consistent with the definition proposed by the 2017 World Workshop on the Classification of Periodontal and Peri‐Implant Diseases and Conditions. While this diagnostic threshold is widely accepted in the literature, recent consensus reports have highlighted that such a highly sensitive dichotomous approach, particularly when based on a single localized bleeding point on probing, may capture minor or transient bleeding events that do not necessarily reflect clinically relevant inflammation [3, 44]. Consequently, this high sensitivity may reduce the ability to detect potential protective effects of keratinized mucosa on peri‐implant mucosal conditions. This potential over‐sensitivity of the diagnostic threshold should therefore be considered when interpreting the absence of a statistically significant association between keratinized mucosa and peri‐implant mucositis in the present meta‐analysis. In contrast, the diagnosis of peri‐implantitis requires radiographic evidence of progressive bone loss, a more objective criterion that is less susceptible to probing‐related artifacts. This difference may explain why a protective association of keratinized mucosa was consistently detected for peri‐implantitis, but not for peri‐implant mucositis, in the present analysis.

It is also important to note that overcontoured restorations or limited access around implants may hinder proper probing and standardized clinical assessment, potentially affecting the detection and interpretation of bleeding on probing.

An important aspect when interpreting the present findings is the interaction between KM width and patient compliance with supportive peri‐implant care (SPC). Previous literature suggests that the protective effect of KM may depend on adherence to maintenance programs. In strictly compliant patients, no significant association between buccal KM width and peri‐implant mucosal inflammation has been observed [45], whereas an adequate KM band (≥ 2 mm) appears to confer protective benefits in erratic compliers [37]. The studies included in our review largely reflect this pattern. For example, Ruiz‐Romero et al. reported that KM < 2 mm was a strong risk indicator for peri‐implantitis in a cohort lacking regular supportive therapy (OR = 5.26), while Costa and Cota identified irregular compliance as a major predictor of peri‐implant disease over an 11‐year follow‐up. Although a number of the included studies accounted for patient compliance levels or recall intervals in multivariable models [24, 26, 28, 30], the substantial heterogeneity in how SPC was defined (e.g., visits per year, regular vs. irregular attendance, or exclusion of compliant patients) precluded a formal stratified meta‐analysis. Future longitudinal studies should standardize SPC reporting and stratify outcomes according to patient compliance to better clarify under which conditions KM width becomes clinically critical.

### 4.4 Risk of Bias and Certainty of Evidence

The risk of bias was conducted using The JBI for Cohort Studies. The high proportion of studies classified as having a low risk of bias (75%) supports the internal validity of the evidence, suggesting that several key methodological domains were adequately addressed across studies. Consistency was observed in exposure measurement across groups (Q2), outcome assessment using valid and reliable methods (Q7), identification of confounding factors (Q4), and sufficient follow‐up duration for outcome manifestation (Q8). Nevertheless, domain‐specific appraisal revealed limitations that warrant cautious interpretation of the results.

Limited reporting on population comparability (Q1), observed in 75% of the studies, represents a relevant limitation, as insufficient baseline characterization may introduce selection bias and hamper the assessment of group comparability. In addition, inadequate handling of attrition and the lack of strategies to address missing data (Q10) emerged as major methodological concerns. Frequent reliance on complete‐case analysis, without imputation or sensitivity analyses, may bias estimates when missingness is related to exposure or outcome.

Regarding statistical analysis, most studies demonstrated acceptable overall adequacy. However, two studies presented limitations in handling of confounding factors. While low‐risk‐of‐bias studies employed multivariable models and rigorous adjustment strategies, studies with moderate and high risk of bias failed to adequately identify confounders or implement appropriate methods to control them. This discrepancy highlights the need for future research to adopt more robust confounding control strategies and advanced regression approaches.

The certainty of evidence, as assessed using the GRADE approach, was rated as low to very low across the evaluated outcomes. This rating was driven primarily by the observational nature of the included studies and by additional limitations related to inconsistency and imprecision in some comparisons, particularly those characterized by substantial heterogeneity and wide confidence intervals. As mentioned before, the observational design of the included studies should not be interpreted as a methodological limitation of this review, but rather as an inherent characteristic of the research question.

The present review was designed to evaluate the association between the presence and inherent width of keratinized mucosa and the risk of peri‐implant diseases. In this context, longitudinal observational studies represent the most appropriate approach to assess disease development over time under natural conditions. Studies evaluating the effects of surgically modifying peri‐implant tissues address a different clinical question and were therefore beyond the scope of the present analysis.

### 4.5 Strengths and Limitations

The main strength of the present systematic review lies in its exclusive focus on longitudinal cohort studies, allowing the assessment of peri‐implant disease incidence rather than prevalence. This approach provides a temporal perspective on disease development and minimizes limitations inherent to cross‐sectional designs and their associated confounders.

An additional strength is the separate evaluation of two clinically relevant keratinized mucosa thresholds (> 0 and ≥ 2 mm), which allows a more nuanced interpretation of the potential role of keratinized mucosa in peri‐implant health. While 2 mm is traditionally adopted as a clinical reference point in the literature, evaluating the 0 mm threshold separately was crucial to isolate the high‐risk scenario represented by the complete absence of tissue. Methodologically, these two thresholds were the only ones providing sufficient data for quantitative synthesis. For studies lacking these specific stratifications, original authors were contacted to retrieve the data. Although a few primary studies explored other cut‐offs (e.g., 1 mm), the limited number of such articles precluded the formation of additional viable meta‐analytical groups.

However, limitations should be considered when interpreting the results. First, all included studies were observational in nature, which inherently limits causal inference. As previously noted, this was an intentional methodological choice, as the research question was not interventional in nature but rather aimed to evaluate the association between the absence or insufficient width of keratinized mucosa and the incidence of peri‐implant diseases under routine clinical conditions. Second, prospective and retrospective cohorts were included, but no statistically significant differences were observed by study design, indicating consistent findings across designs. This suggests that differences in point estimates may reflect variation in event rates and limited statistical power rather than effect differences.

It should be acknowledged that the < 2 mm category may have included sites with complete absence of keratinized mucosa (0 mm), which may have influenced the observed estimates. However, the separate evaluation of the thresholds (> 0 mm vs. 0 mm and ≥ 2 mm vs. < 2 mm) still allows a more nuanced interpretation of the potential role of KM width. The slightly stronger association observed for the ≥ 2 mm threshold, compared with the mere presence of KM, may be compatible with a possible additional benefit of wider bands of keratinized mucosa, although this interpretation should be made cautiously. Importantly, the categorical reporting of the included cohort studies did not allow sites with 0 mm KM to be distinguished within the < 2 mm group.

A limitation of the current evidence is that KM assessment was predominantly performed at the buccal aspect, with limited reporting of lingual sites in the primary literature. Furthermore, because the included studies reported peri‐implant disease incidence as a dichotomous outcome at the implant level, site‐specific analyses correlating localized KM width with adjacent inflammatory or destructive lesions were not feasible. This limitation reflects the structure of the available evidence rather than the analytical approach of the present review. Another limitation of the current evidence is the limited and inconsistent reporting of relevant anatomical, surgical, and prosthetic‐related parameters in the included cohort studies, many of which may influence both keratinized mucosa dimensions and peri‐implant tissue stability. These factors include, for example, implant positioning, the apico‐coronal position of the implant shoulder relative to the bone crest, the depth of the transmucosal tunnel, implant malpositioning, and the presence and extent of bone dehiscences [46, 47, 48]. Because these variables were not assessed or consistently reported across studies, their potential confounding effect could not be adequately evaluated, which limits the interpretation of the independent role of keratinized mucosa in the development of peri‐implant diseases. Furthermore, while the included studies frequently reported overall prosthetic features at the cohort level, they did not cross‐stratify event data by these variables (e.g., unitary versus multiple restorations) and keratinized mucosa thresholds. Consequently, subgroup meta‐analyses based on specific prosthetic configurations were unfeasible.

Another methodological consideration relates to the potential clustering of implants within the same patient. Although patient‐level data are generally more robust for accounting for systemic risk factors, when multiple implants per individual are analyzed without considering intra‐subject correlation, the assumption of independence may be violated, potentially leading to underestimated variance and narrower confidence intervals, particularly when pooling implant‐level data across studies. However, it is important to note that several included studies accounted for clustering using multilevel or GEE‐based models, although this was not consistently addressed across all studies and could not be accounted for at the meta‐analytic level.

A further limitation of the present meta‐analysis is the reliance on crude implant‐level event counts rather than adjusted effect estimates. Keratinized mucosa is not randomly distributed and may be associated with other important determinants of peri‐implant health, such as history of periodontitis, smoking, and maintenance compliance. Therefore, these factors may act as confounders in the observed association between keratinized mucosa and peri‐implant outcomes. Indeed, systemic conditions such as smoking and a history of periodontitis have consistently been identified as significant risk indicators for peri‐implant disease. For instance, recent meta‐analyses of prospective cohort studies have shown that smokers present approximately twice the risk of developing peri‐implantitis compared with non‐smokers [49]. Similarly, patients with a history of periodontitis show a substantially higher incidence of peri‐implantitis and implant loss over time [50]. Given that the protective effect of KM may be particularly relevant in erratic compliers, irregular maintenance can further amplify disease risk at sites lacking KM [37, 45]. Although several primary studies performed multivariable analyses, a meta‐analysis of adjusted estimates was not feasible due to selective reporting. In some cohorts, KM was excluded from the final multivariable models when it was not statistically significant, resulting in no adjusted estimates being reported. Therefore, the pooled results based on crude data should be interpreted cautiously, as the observed association between lack of KM and peri‐implantitis may be partially influenced by residual confounding and should therefore be interpreted with caution.

Furthermore, the treatment of incidence in our meta‐analysis requires careful interpretation. Due to the lack of time‐to‐event data or incidence rates (person‐years) reported in the primary studies, our analysis relied on pooling cumulative risks across heterogeneous follow‐up windows. To mitigate this, we performed stratified analyses based on follow‐up duration (≤ 5 and > 5 years). Additionally, it is important to acknowledge that the inclusion of retrospective cohort studies, which are often based on a single recall several years post‐implant placement, carries inherent uncertainty regarding the exact timing of disease onset when compared to prospective surveillance.

The way missing data and incomplete follow‐up were handled across the primary studies also warrants attention, since non‐attenders were predominantly excluded from the analyses (Table 5). Because patients lost to follow‐up may have poorer oral hygiene and less regular compliance with supportive peri‐implant care, this approach introduces the potential for attrition bias. As previously discussed, this issue may be particularly relevant when interpreting the effect of keratinized mucosa, since the protective role of KM may be more evident in patients with less favorable maintenance behavior. Therefore, the exclusion of these individuals may have influenced the observed estimates and should be considered when interpreting the pooled findings.

Finally, substantial heterogeneity was observed in some analyses, likely reflecting variability in study populations, follow‐up duration, and diagnostic criteria. Differences in the definitions of peri‐implant diseases may have affected the sensitivity for detecting disease, whereas longer follow‐up periods inherently increase the likelihood of identifying incident cases. These sources of heterogeneity could not be fully accounted for in quantitative analyses due to inconsistent reporting across studies. These factors act independently and represent well‐recognized challenges in the synthesis of incidence data, particularly in fields where longitudinal evidence remains limited.

Despite these limitations, the present findings provide clinically relevant insights into the role of keratinized mucosa as a potential protective factor against peri‐implantitis and highlight important gaps to be addressed by future well‐designed longitudinal studies.

### 4.6 Implications for Clinical Practice and Future Research

- From a clinical perspective, the present findings indicate that the presence and width of keratinized mucosa should be considered in peri‐implant risk assessment. Reduced or absent keratinized mucosa was associated with a higher risk of peri‐implantitis, suggesting that these sites may require closer clinical attention. Given the low certainty of evidence, reduced or absent keratinized mucosa should be primarily emphasized as a risk indicator for surveillance rather than implying a direct causal or interventional recommendation.
- In practical terms, assessment of keratinized mucosa may help clinicians individualize peri‐implant care strategies. Depending on the overall risk profile, this may include tailored maintenance protocols with shorter recall intervals, reinforced oral hygiene measures, or consideration of additional therapeutic approaches, such as peri‐implant phenotype modification.
- From a research perspective, future longitudinal studies should combine detailed reporting of peri‐implant soft tissue conditions with standardized follow‐up and maintenance protocols to improve the interpretation of long‐term outcomes and adopt standardized outcome measures that capture peri‐implant inflammation beyond bleeding on probing alone.
- Greater standardization in disease definitions, follow‐up protocols, reporting of soft tissue parameters, and handling of confounding and missing data will be essential to advance evidence‐based personalization of peri‐implant care.

## 5 Conclusions

### 5.1 Implications for Clinical Practice

Evidence from cohort studies suggests no association between keratinized mucosa and the incidence of peri‐implant mucositis. In contrast, implants with keratinized mucosa and those with a width ≥ 2 mm showed a lower incidence of peri‐implantitis. Within the limitations of the available evidence, keratinized mucosa should therefore be considered as a risk indicator for surveillance in peri‐implant risk assessment.

### 5.2 Implications for Research

Further well‐designed longitudinal cohort studies using standardized diagnostic criteria, a more comprehensive assessment of keratinized mucosa, systematic reporting of relevant anatomical, surgical, and prosthetic factors, and better control of confounding variables are warranted to strengthen the certainty of the evidence. Relevant anatomical and surgical factors include bone characteristics; the presence and extent of bone dehiscences (i.e., presence/absence and, when present, the extent of buccal and/or lingual bone dehiscence around the implant; e.g., assessed intraoperatively or by 3D imaging); implant malpositioning (i.e., deviation from the ideal prosthetic position in three dimensions, such as excessively buccal placement; e.g., evaluated by 3D imaging); the apico‐coronal position of the implant shoulder relative to the bone crest (i.e., the vertical relationship of the implant platform to the adjacent alveolar bone; e.g., measured on standardized radiographs); and implant type (e.g., tissue‐level vs. bone‐level design). Prosthetic‐related factors include the depth of the transmucosal tunnel (i.e., the vertical distance from the mucosal margin to the implant platform; e.g., measured clinically with a periodontal probe), as well as prosthetic and abutment configuration. In addition to implant‐level analyses, patient‐level analyses should also be encouraged to better account for individual risk profiles and the clustering of implants within the same patient.

## Funding

The authors have nothing to report.

## Supporting information


**Table S1:** Search strategies used for each electronic database.
**Table S2:** Reasons for exclusions of 386 papers.
**Table S3:** Complete Risk of bias assessment according to the Joanna Briggs Institute for Cohort Studies and reasons.
**Table S4:** GRADE summary of findings.

**Figure S1:** Forest plot for peri‐implant mucositis comparing present (> 0 mm) vs. absent (0 mm) keratinized mucosa, with subgroup analysis by follow‐up duration (≤ 5 years vs. > 5 years).
**Figure S2:** Forest plot for peri‐implant mucositis comparing ≥ 2 mm vs. < 2 mm of keratinized mucosa, with subgroup analysis by follow‐up duration (≤ 5 years vs. > 5 years).
**Figure S3:** Forest plot for peri‐implantitis comparing present (> 0 mm) vs. absent (0 mm) keratinized mucosa, with subgroup analysis by follow‐up duration (≤ 5 years vs. > 5 years).
**Figure S4:** Forest plot for peri‐implant mucositis comparing ≥ 2 mm vs. < 2 mm of keratinized mucosa, with subgroup analysis by follow‐up duration (≤ 5 years vs. > 5 years).
**Figure S5:** Forest plot for peri‐implantitis comparing present (> 0 mm) vs. absent (0 mm) keratinized mucosa, with subgroup analysis by peri‐implantitis case definition thresholds (marginal bone loss and probing depth).
**Figure S6:** Forest plot for peri‐implantitis comparing 2 mm vs. < 2 mm of keratinized mucosa, with subgroup analysis by peri‐implantitis case definition thresholds (marginal bone loss and probing depth).

## Data Availability

The data that supports the findings of this study are available in the Tables S1–S4 and Figures S1–S6.
