# Supplementary material for: Keratinized Mucosa Width and Incidence of Peri‐Implant Diseases: A Systematic Review With Meta‐Analysis
Source: J Periodontal Res. 2026 May 14;61(5):452–72. doi: 10.1111/jre.70123 (PMC13378225; doi:10.1111/jre.70123)
Supplement: Supplementary file 1 — Table S1: Search strategies used for each electronic database. Table S2: Reasons for exclusions of 386 papers. Table S3: Complete Risk of bias assessment according to the Joanna Briggs Institute for Cohort Studies and reasons. Table S4: GRADE summary of findings. [file JRE-61-452-s002.docx]

**Supplementary Table 1.** Search strategies used for each electronic database

| Search strategies | |
| --- | --- |
| **PubMed** | #1 ((((((((Peri-implantitis[MeSH Terms]) OR (periimplantitis)) OR ("peri-implant disease*")) OR ("periimplant disease*")) OR ("peri-implant inflammat*")) OR (peri-implant mucositis)) OR (periimplant mucositis)) OR ("biologic complication*")) OR ("peri-implant patholog*")  #2 (((((keratinized) OR (kt)) OR (attached)) OR ("tissue phenotype")) OR ("tissue width")) OR ("soft tissue")  #1 AND #2 |
| **Embase** | ('periimplantitis'/exp OR 'peri-implant mucositis'/exp OR 'implant complication'/exp) AND ('keratinized tissue width'/exp OR 'attached' OR 'soft tissue'/exp) |
| **Web of science** | #1 peri-implantitis OR peri-implant mucositis OR peri-implant disease*  #2 keratinized OR attached OR soft tissue  #1 AND #2 |
| **Scopus** | peri-implantitis OR peri-implant AND mucositis AND keratinized OR attached |

**Supplementary Table 2.** Reasons for exclusions of 386 papers

| Reference | Reasons for exclusions |
| --- | --- |
| 1. Able et al., 2021 | No data about KM |
| 1. AbuHussien et al., 2023 | No data about KM |
| 1. Aguirre-Zorzano et al., 2013 | No data about KM |
| 1. Agustín-Panadero et al., 2019 | No data about PI and/or PIM |
| 1. Şahin et al., 2023 | No data about KM |
| 1. Akça et al., 2010 | No data about PI and/or PIM |
| 1. Alami et al., 2024 | No data about KM |
| 1. AlAmri et al., 2017 | No data about PI and/or PIM |
| 1. AlAmri et al., 2017 | No data about PI and/or PIM |
| 1. Alan et al., 2015 | No data about PI and/or PIM |
| 1. Alasqah et al., 2018 | No data about PI and/or PIM |
| 1. Alawad et al., 2023 | No data about PI and/or PIM |
| 1. Alharthi et al., 2018 | No data about PI and/or PIM |
| 1. AlHelal et al., 2024 | No data about PI and/or PIM |
| 1. AlJasser et al., 2021 | No data about KM |
| 1. Alkhudhairy et al., 2018 | No data about PI and/or PIM |
| 1. Almahrous et al., 2020 | No data about KM |
| 1. Askin et al., 2015 | No data about KM |
| 1. Alrabiah et al., 2019 | No data about PI and/or PIM |
| 1. Alshahrani et al., 2019 | No data about PI and/or PIM |
| 1. Al-Sowygh et al., 2018 | No data about PI and/or PIM |
| 1. Anitua et al., 2016 | No data about PI and/or PIM |
| 1. Apaza-Bedoya et al., 2024 | No data about KM |
| 1. Asgeirsson et al., 2019 | No data about KM |
| 1. Asik et al., 2024 | No data about PI and/or PIM |
| 1. Asik et al., 2015 | No data about PI and/or PIM |
| 1. Atalay et al., 2013 | No data about PI and/or PIM |
| 1. Awaad et al., 2023 | No data about KM |
| 1. Axiotis et al., 2018 | No data about PI and/or PIM |
| 1. Ayuningtyas et al., 2023 | No data about PI and/or PIM |
| 1. Babayiğit et al., 2024 | No data about PI and/or PIM |
| 1. Bae et al., 2017 | No data about PI and/or PIM |
| 1. Begić et al., 2025 | No data about KM |
| 1. Bekcioglu et al., 2012 | No data about KM |
| 1. Benedek et al., 2024 | No data about KM |
| 1. Bengazi et al., 1996 | No data about KM |
| 1. Benic et al., 2009 | No data about PI and/or PIM |
| 1. Beschnidt et al., 2018 | No data about KM |
| 1. Betthäuser et al., 2022 | No data about KM |
| 1. Bhat et al., 2015 | No data about PI and/or PIM |
| 1. Bianchini et al., 2024 | No data about PI and/or PIM |
| 1. Bienz et al., 2023 | No data about KM |
| 1. Bilhan et al., 2021 | No data about PI and/or PIM |
| 1. Blake et al., 2008 | No data about KM |
| 1. Blume et al., 2020 | No data about KM |
| 1. Bollen et al., 1996 | No data about KM |
| 1. Borre et al., 2024 | No data about KM |
| 1. Boynuegri et al., 2013 | No data about PI and/or PIM |
| 1. Brägger et al., 1997 | No data about KM |
| 1. Breunig et al., 2024 | No data about KM |
| 1. Baumer et al., 2017 | No data about KM |
| 1. Buser et al., 2012 | No data about KM |
| 1. Caccianiga et al., 2021 | No data about KM |
| 1. Cairo et al., 2020 | No data about KM |
| 1. Camarda et al., 2020 | No data about KM |
| 1. Cambiaghi et al., 2024 | No data about KM |
| 1. Candel-Marti et al., 2015 | No data about KM |
| 1. Canullo et al., 2016 | Case Reports/Case Series/ Reviews/ RCTs/Cross-Sectional Studies |
| 1. Canullo et al., 2007 | No data about PI and/or PIM |
| 1. Cappare et al., 2021 | Not found in any electronic databases |
| 1. Carral et al., 2021 | No data about PI and/or PIM |
| 1. Carvalhaes et al., 2024 | No data about PI and/or PIM |
| 1. Casula et al., 2021 | No data about KM |
| 1. Ceruso et al., 2021 | No data about PI and/or PIM |
| 1. Changi et al., 2019 | No data about PI and/or PIM |
| 1. Chang et al., 2013 | No data about PI and/or PIM |
| 1. Chang et al., 2010 | No data about KM |
| 1. Chang et al., 2011 | No data about PI and/or PIM |
| 1. Cheung et al., 2021 | No data about KM |
| 1. Chochlidakis et al., 2022 | No data about KM |
| 1. Chung et al., 2007 | No data about PI and/or PIM |
| 1. Clauser et al., 2020 | No data about KM |
| 1. Cobo-Vázquez et al., 2025 | No data about PI and/or PIM |
| 1. Cosyn et al., 2013 | No data about PI and/or PIM |
| 1. Covani et al., 2004 | No data about PI and/or PIM |
| 1. Crespi et al., 2019 | No data about PI and/or PIM |
| 1. Crespi et al., 2024 | No data about KM |
| 1. Cucchi et al., 2023 | No data about KM |
| 1. Custódio et al., 2024 | No data about PI and/or PIM |
| 1. Dalago et al., 2017 | Case Reports/Case Series/ Reviews/ rcts/Cross-Sectional Studies |
| 1. Danser et al., 1997 | No data about KM |
| 1. Dash et al., 2024 | No data about PI and/or PIM |
| 1. DeAngelo et al., 2007 | No data about PI and/or PIM |
| 1. deAraújoNobre et al., 2024 | No data about PI and/or PIM |
| 1. deAraújoNobre et al., 2015 | No data about PI and/or PIM |
| 1. Degidi et al., 2010 | No data about PI and/or PIM |
| 1. deKok et al., 2006 | No data about PI and/or PIM |
| 1. delCastillo et al., 2023 | Case Reports/Case Series/ Reviews/ rcts/Cross-Sectional Studies |
| 1. deMedeirosDantas et al., 2024 | No data about KM |
| 1. deMelo et al., 2017 | No data about KM |
| 1. Derks et al., 2016 | No data about KM |
| 1. DeRy et al., 2021 | No data about KM |
| 1. deSiqueira et al., 2020 | No data about KM |
| 1. deVasconcelosGurgel et al., 2021 | No data about KM |
| 1. Duque et al., 2016 | No data about KM |
| 1. Ebadian et al., 2020 | No data about PI and/or PIM |
| 1. Ekfeldt et al., 2017 | No data about KM |
| 1. Elemek et al., 2020 | Case Reports/Case Series/ Reviews/ RCTs/Cross-Sectional Studies |
| 1. Esfahanizadeh et al., 2016 | No data about PI and/or PIM |
| 1. Esper et al., 2012 | No data about PI and/or PIM |
| 1. Esposito et al., 2017 | No data about KM |
| 1. Fang et al., 2012 | No data about PI and/or PIM |
| 1. Farzad et al., 2004 | No data about PI and/or PIM |
| 1. Fernandes-Costa et al., 2019 | No data about KM |
| 1. Ferreira et al., 2015 | Case Reports/Case Series/ Reviews/ rcts/Cross-Sectional Studies |
| 1. Ferreira et al., 2006 | No data about KM |
| 1. Finne et al., 2012 | No data about KM |
| 1. French et al., 2024 | No data about PI and/or PIM |
| 1. French et al., 2016 | No data about PI and/or PIM |
| 1. French et al., 2019 | No data about KM |
| 1. French et al., 2018 | No data about KM |
| 1. French et al., 2019 | No data about PI and/or PIM |
| 1. Frisch et al., 2019 | Not found in any electronic databases |
| 1. Frisch et al., 2020 | No data about KM |
| 1. Frisch et al., 2013 | No data about KM |
| 1. Frisch et al., 2015 | No data about KM |
| 1. Galindo-Moreno et al., 2023 | No data about PI and/or PIM |
| 1. Galindo-Moreno et al., 2014 | No data about PI and/or PIM |
| 1. Gallucci et al., 2009 | No data about PI and/or PIM |
| 1. Gao et al., 2024 | Not found in any electronic databases |
| 1. Garaicoa-Pazmino et al., 2021 | No data about PI and/or PIM |
| 1. Gehrke et al., 2023 | No data about KM |
| 1. Genetti et al., 2021 | No data about PI and/or PIM |
| 1. Gharpure et al., 2021 | Case Reports/Case Series/ Reviews/ rcts/Cross-Sectional Studies |
| 1. Giralt-Hernando et al., 2022 | No data about PI and/or PIM |
| 1. Glauser et al., 2016 | No data about KM |
| 1. Güler et al., 2021 | No data about PI and/or PIM |
| 1. Glibert et al., 2016 | No data about PI and/or PIM |
| 1. Golob et al., 2024 | No data about KM |
| 1. Gosai et al., 2022 | No data about KM |
| 1. Grischke et al., 2019 | No data about KM |
| 1. Grischke et al., 2021 | No data about KM |
| 1. Gualini et al., 2003 | No data about KM |
| 1. Guarnieri et al., 2018 | No data about KM |
| 1. Guarnieri et al., 2015 | No data about PI and/or PIM |
| 1. Guarnieri et al., 2018 | No data about PI and/or PIM |
| 1. Guarnieri et al., 2019 | No data about PI and/or PIM |
| 1. Guarnieri et al., 2019 | No data about PI and/or PIM |
| 1. Guarnieri et al., 2018 | No data about KM |
| 1. Guarnieri et al., 2014 | No data about PI and/or PIM |
| 1. Guarnieri et al., 2016 | No data about PI and/or PIM |
| 1. Guarnieri et al., 2020 | No data about KM |
| 1. Gunpinar et al., 2020 | No data about KM |
| 1. Gurbuz et al., 2024 | No data about PI and/or PIM |
| 1. Gurbuz et al., 2023 | No data about PI and/or PIM |
| 1. Hall et al., 2019 | No data about PI and/or PIM |
| 1. Halperin et al., 2016 | No data about PI and/or PIM |
| 1. Hamudi et al., 2022 | No data about KM |
| 1. Hashim et al., 2020 | No data about PI and/or PIM |
| 1. Heller et al., 2023 | No data about PI and/or PIM |
| 1. Heng et al., 2024 | No data about KM |
| 1. Heschl et al., 2013 | No data about PI and/or PIM |
| 1. Hessling et al., 2015 | No data about KM |
| 1. Hinze et al., 2013 | No data about PI and/or PIM |
| 1. Hoerler et al., 2024 | No data about PI and/or PIM |
| 1. Horikawa et al., 2017 | No data about PI and/or PIM |
| 1. Hussain et al., 2024 | No data about KM |
| 1. Hu et al., 2014 | No data about PI and/or PIM |
| 1. Ibraheem et al., 2022 | No data about PI and/or PIM |
| 1. Iglhaut et al., 2021 | No data about KM |
| 1. Isler et al., 2022 | No data about PI and/or PIM |
| 1. Isler et al., 2024 | No data about KM |
| 1. Isler et al., 2018 | No data about KM |
| 1. Jasser et al., 2021 | No data about KM |
| 1. Jeong et al., 2011 | No data about KM |
| 1. Jepsen et al., 1996 | No data about KM |
| 1. Jervøe-Storm et al., 2023 | No data about KM |
| 1. Jungner et al., 2014 | No data about PI and/or PIM |
| 1. Jung et al., 2013 | No data about PI and/or PIM |
| 1. Kadkhodazadeh et al., 2024 | No data about KM |
| 1. Kadkhodazadeh et al., 2022a | No data about KM |
| 1. Kadkhodazadeh et al., 2022b | No data about PI and/or PIM |
| 1. Kandaswamy et al., 2022 | No data about KM |
| 1. Kaptein et al., 1999 | No data about PI and/or PIM |
| 1. Karcı et al., 2021 | No data about KM |
| 1. Karoussis et al., 2003 | No data about KM |
| 1. Khalifah et al., 2024 | No data about KM |
| 1. Khatavkar et al., 2021 | Not found in any electronic databases |
| 1. Kikuchi et al., 2022 | No data about KM |
| 1. Kim et al., 2009 | No data about KM |
| 1. Kim et al., 2025 | No data about PI and/or PIM |
| 1. Kim et al., 2015 | No data about PI and/or PIM |
| 1. Kiran et al., 2018 | No data about PI and/or PIM |
| 1. Kissa et al., 2021 | No data about KM |
| 1. Kniha et al., 2018 | No data about PI and/or PIM |
| 1. Kohal et al., 2018 | No data about PI and/or PIM |
| 1. Kohal et al., 2023 | No data about KM |
| 1. Konstantinidis et al., 2015 | No data about KM |
| 1. Kozakiewicz et al., 2022 | No data about KM |
| 1. Krebs et al., 2024 | No data about KM |
| 1. Krennmair et al., 2010 | No data about PI and/or PIM |
| 1. Krennmair et al., 2023 | No data about KM |
| 1. Krennmair et al., 2019 | No data about KM |
| 1. Krennmair et al., 2016 | No data about KM |
| 1. Kungsadalpipob et al., 2020 | Case Reports/Case Series/ Reviews/ rcts/Cross-Sectional Studies |
| 1. Kwon et al., 2010 | No data about KM |
| 1. Ladwein et al., 2015 | No data about KM |
| 1. Lamperti et al., 2022 | No data about KM |
| 1. Landes et al., 2012 | No data about KM |
| 1. Lee et al., 2023 | No data about KM |
| 1. Lehmann et al., 2013 | No data about KM |
| 1. Leone et al., 2024 | No data about KM |
| 1. Lim et al., 2019 | No data about KM |
| 1. Lin et al., 2023 | Case Reports/Case Series/ Reviews/ rcts/Cross-Sectional Studies |
| 1. Liu et al., 2019 | No data about KM |
| 1. Lombardo et al., 2014 | No data about KM |
| 1. Lombardo et al., 2020 | No data about KM |
| 1. Lombardo et al., 2022 | No data about PI and/or PIM |
| 1. Lorenz et al., 2019 | No data about KM |
| 1. Lorenz et al., 2024 | Case Reports/Case Series/ Reviews/ rcts/Cross-Sectional Studies |
| 1. Lorenz et al., 2019 | No data about KM |
| 1. Lorenz et al., 2017 | No data about KM |
| 1. Lorenz et al., 2022 | No data about KM |
| 1. Mailoa et al., 2015 | No data about KM |
| 1. Majid et al., 2024 | Not found in any electronic databases |
| 1. Malchiodi et al., 2015 | No data about KM |
| 1. Maló et al., 2013 | No data about PI and/or PIM |
| 1. Mameno et al., 2019 | No data about KM |
| 1. Mameno et al., 2020 | No data about PI and/or PIM |
| 1. Mangano et al., 2015 | No data about KM |
| 1. Manopattanasoontorn et al., 2021 | No data about PI and/or PIM |
| 1. Marcantonio et al., 2021 | No data about KM |
| 1. Mareque-Bueno et al., 2011 | No data about PI and/or PIM |
| 1. Matarazzo et al., 2018 | No data about KM |
| 1. Mei et al., 2017 | No data about KM |
| 1. Menini et al., 2017 | No data about PI and/or PIM |
| 1. Menini et al., 2015 | No data about PI and/or PIM |
| 1. Menini et al., 2022 | No data about PI and/or PIM |
| 1. Menini et al., 2018 | No data about KM |
| 1. Mericske-Stern et al., 1994 | No data about PI and/or PIM |
| 1. Meyle et al., 2014 | No data about KM |
| 1. Michels et al., 2022 | Not found in any electronic databases |
| 1. Kayal et al., 2024 | No data about PI and/or PIM |
| 1. Müller et al., 2015 | No data about KM |
| 1. Mo et al., 2022 | No data about KM |
| 1. Monje et al., 2019 | No data about KM |
| 1. Monje et al., 2018 | No data about KM |
| 1. Monje et al., 2021 | No data about KM |
| 1. Monje et al., 2017 | No data about KM |
| 1. Montaruli et al., 2023 | No data about PI and/or PIM |
| 1. Mumcu et al., 2019 | No data about PI and/or PIM |
| 1. Muñoz-Cámara et al., 2020 | No data about PI and/or PIM |
| 1. Naeini et al., 2023 | No data about KM |
| 1. Nagni et al., 2024 | No data about PI and/or PIM |
| 1. Nicoli et al., 2017 | No data about KM |
| 1. Norton et al., 2023 | No data about PI and/or PIM |
| 1. Närhi et al., 2001 | No data about PI and/or PIM |
| 1. Obreja et al., 2022 | No data about KM |
| 1. Ogata et al., 2017 | No data about KM |
| 1. Oliveira et al., 2023 | Implants with machined (smooth) surfaces |
| 1. Onclin et al., 2023 | No data about KM |
| 1. Ortiz-Echeverri et al., 2024 | No data about KM |
| 1. Ow et al., 1999 | No data about PI and/or PIM |
| 1. Paken et al., 2021 | No data about KM |
| 1. Pamato et al., 2020 | No data about KM |
| 1. Papalou et al., 2022 | No data about KM |
| 1. Papaspyridakos et al., 2018 | No data about KM |
| 1. Papaspyridakos et al., 2019 | No data about KM |
| 1. Park et al., 2017 | No data about PI and/or PIM |
| 1. Parpaiola et al., 2015 | No data about PI and/or PIM |
| 1. Parpaiola et al., 2024 | No data about PI and/or PIM |
| 1. Parvini et al., 2022 | No data about PI and/or PIM |
| 1. Parvini et al., 2020 | No data about KM |
| 1. Parvini et al., 2023 | No data about KM |
| 1. Passoni et al., 2014 | No data about KM |
| 1. Peñarrocha-Oltra et al., 2013 | No data about KM |
| 1. Pelekos et al., 2023 | No data about KM |
| 1. Pellicer-Chover et al., 2019 | No data about KM |
| 1. Pellicer-Chover et al., 2014 | No data about KM |
| 1. Perti et al., 2023 | No data about PI and/or PIM |
| 1. Perussolo et al., 2022 | No data about KM |
| 1. Perussolo et al., 2018 | No data about KM |
| 1. Pieralli et al., 2021 | No data about PI and/or PIM |
| 1. Pimentel et al., 2018 | No data about KM |
| 1. Pjetursson et al., 2012 | No data about KM |
| 1. Poli et al., 2023 | No data about KM |
| 1. Pozzi et al., 2023 | No data about KM |
| 1. Pozzi et al., 2016 | No data about KM |
| 1. Puisys et al., 2024 | No data about PI and/or PIM |
| 1. Oliveira et al., 2023 | Implants with machined (smooth) surfaces |
| 1. Qian et al., 2018 | No data about PI and/or PIM |
| 1. Queridinha et al., 2016 | No data about KM |
| 1. Quispe-López et al., 2024 | No data about PI and/or PIM |
| 1. Raabe et al., 2021 | No data about KM |
| 1. Ragucci et al., 2020 | No data about PI and/or PIM |
| 1. Raj et al., 2022 | No data about PI and/or PIM |
| 1. Rakasevic et al., 2022 | No data about KM |
| 1. Ramananda et al., 2022 | No data about PI and/or PIM |
| 1. Rammelsberg et al., 2017 | No data about KM |
| 1. Rathi et al., 2022 | No data about KM |
| 1. Ravald et al., 2013 | No data about KM |
| 1. Rekawek et al., 2021 | No data about KM |
| 1. Ren et al., 2017 | No data about KM |
| 1. Rinke et al., 2020 | Case Reports/Case Series/ Reviews/ rcts/Cross-Sectional Studies |
| 1. Roccuzzo et al., 2024 | No data about KM |
| 1. Roccuzzo et al., 2023 | No data about KM |
| 1. Roccuzzo et al., 2014 | No data about PI and/or PIM |
| 1. Roccuzzo et al., 2016 | No data about PI and/or PIM |
| 1. Rodrigo et al., 2018 | No data about KM |
| 1. Rodriguez et al., 2022 | No data about KM |
| 1. Rokn et al., 2017 | No data about KM |
| 1. Romandini et al., 2021 | Case Reports/Case Series/ Reviews/ RCTs/Cross-Sectional Studies |
| 1. Romandini et al., 2021 | No data about KM |
| 1. Romandini et al., 2021 | No data about KM |
| 1. Romanos et al., 2023 | No data about PI and/or PIM |
| 1. Romanos et al., 2015 | No data about PI and/or PIM |
| 1. Roos-Jansåker et al., 2006 | Implants with machined (smooth) surfaces |
| 1. Rothamel et al., 2022 | No data about KM |
| 1. Rungtanakiat et al., 2023 | No data about KM |
| 1. Rungtanakiat et al., 2024 | No data about KM |
| 1. Sanz-Sánchez et al., 2024 | No data about KM |
| 1. Schiegnitz et al., 2017 | No data about PI and/or PIM |
| 1. Schmidt et al., 2019 | No data about PI and/or PIM |
| 1. Schmitt et al., 2014 | No data about PI and/or PIM |
| 1. Schropp et al., 2014 | No data about KM |
| 1. Schrott et al., 2009 | No data about PI and/or PIM |
| 1. Schuldt Filho et al., 2014 | No data about KM |
| 1. Schwartzenberg et al., 2022 | No data about KM |
| 1. Schwarz et al., 2018 | No data about KM |
| 1. Schwarz et al., 2017 | Case Reports/Case Series/ Reviews/ rcts/Cross-Sectional Studies |
| 1. Sekundo et al., 2020 | No data about KM |
| 1. Seyssens et al., 2020 | No data about KM |
| 1. Shah et al., 2017 | No data about PI and/or PIM |
| 1. Shatta et al., 2019 | No data about KM |
| 1. Shi et al., 2022 | No data about PI and/or PIM |
| 1. Shimomoto et al., 2021 | No data about PI and/or PIM |
| 1. Slutzkey et al., 2022 | No data about KM |
| 1. Souza et al., 2016 | No data about PI and/or PIM |
| 1. Östman et al., 2012 | No data about KM |
| 1. Sukuroglu et al., 2019 | No data about PI and/or PIM |
| 1. Suzuki et al., 2024 | No data about PI and/or PIM |
| 1. Tabrizi et al., 2024 | No data about PI and/or PIM |
| 1. Takahashi et al., 2018 | No data about PI and/or PIM |
| 1. Tang et al., 2000 | No data about PI and/or PIM |
| 1. Tastan Eroglu et al., 2024 | No data about PI and/or PIM |
| 1. Tavelli et al., 2022 | No data about PI and/or PIM |
| 1. Tawse-Smith et al., 2017 | No data about PI and/or PIM |
| 1. Tenenbaum et al., 2017 | No data about KM |
| 1. Tlustenko et al., 2021 | No data about KM |
| 1. Todisco et al., 2019 | No data about PI and/or PIM |
| 1. Toia et al., 2024 | No data about KM |
| 1. Toia et al., 2022 | No data about KM |
| 1. Toljanic et al., 2001 | No data about PI and/or PIM |
| 1. Tomina et al., 2023 | No data about KM |
| 1. Tormena et al., 2020 | No data about PI and/or PIM |
| 1. Torul et al., 2021 | No data about KM |
| 1. Trombelli et al., 2020 | No data about PI and/or PIM |
| 1. Tur et al., 2023 | No data about PI and/or PIM |
| 1. Ueno et al., 2016 | No data about PI and/or PIM |
| 1. Urban et al., 2012 | No data about PI and/or PIM |
| 1. Van Brakel et al., 2012 | No data about PI and/or PIM |
| 1. Van Velzen et al., 2015 | No data about KM |
| 1. Vervaeke et al., 2014 | No data about PI and/or PIM |
| 1. Vianna et al., 2018 | No data about PI and/or PIM |
| 1. Vignoletti et al., 2019 | No data about KM |
| 1. Vigolo et al., 2016 | No data about PI and/or PIM |
| 1. Vilarrasa et al., 2021 | Case Reports/Case Series/ Reviews/ rcts/Cross-Sectional Studies |
| 1. Vilhjálmsson et al., 2013 | No data about KM |
| 1. Volp Junior et al., 2024 | No data about KM |
| 1. Wada et al., 2019 | Case Reports/Case Series/ Reviews/ rcts/Cross-Sectional Studies |
| 1. Wahab et al., 2023 | No data about KM |
| 1. Wang et al., 2020 | No data about PI and/or PIM |
| 1. Wei et al., 2024 | No data about PI and/or PIM |
| 1. Wennström et al., 1994 | No data about PI and/or PIM |
| 1. Wessels et al., 2020 | No data about KM |
| 1. Widbom et al., 2005 | No data about KM |
| 1. Wiesli et al., 2023 | No data about KM |
| 1. Wilson et al., 2019 | No data about PI and/or PIM |
| 1. Windael et al., 2021 | No data about KM |
| 1. Windael et al., 2024 | No data about KM |
| 1. Windael et al., 2018 | No data about KM |
| 1. Woelber et al., 2016 | No data about KM |
| 1. Wu et al., 2020 | No data about KM |
| 1. Wu et al., 2023 | No data about KM |
| 1. Xu et al., 2024 | No data about KM |
| 1. Yang et al., 2024 | No data about PI and/or PIM |
| 1. Yang et al., 2023 | No data about KM |
| 1. Yeung et al., 2008 | No data about PI and/or PIM |
| 1. Yilihamujiang et al., 2024 | No data about KM |
| 1. Yoo et al., 2021 | No data about KM |
| 1. Yoo et al., 2021 | No data about KM |
| 1. Zahng et al., 2021 | No data about KM |
| 1. Zetterqvist et al., 2010 | No data about KM |
| 1. Zhang et al., 2016 | No data about PI and/or PIM |
| 1. Zhang et al., 2020 | Case Reports/Case Series/ Reviews/ rcts/Cross-Sectional Studies |
| 1. Zhang et al., 2020 | No data about KM |
| 1. Zhao et al., 2016 | No data about KM |
| 1. Zhou et al., 2024 | No data about PI and/or PIM |
| 1. Zigdon et al., 2008 | No data about PI and/or PIM |
| 1. Zitzmann et al., 2001 | No data about PI and/or PIM |
| 1. Zuercher et al., 2022 | No data about PI and/or PIM |

**Supplementary Table 3.** Complete Risk of bias assessment according to the Joanna Briggs Institute for Cohort Studies and reasons.

| **Studies** | **Q1** | R | **Q2** | R | **Q3** | R | **Q4** | R | **Q5** | R | **Q6** | R | **Q7** | R | **Q8** | R | **Q9** | R | **Q10** | R | **Q11** | R |
| --- | --- | --- | --- | --- | --- | --- | --- | --- | --- | --- | --- | --- | --- | --- | --- | --- | --- | --- | --- | --- | --- | --- |
| Alhakeem2023 | U | No table or description of baseline prognostic factors between KT ≥2 mm and KT <2 mm groups was provided. | Y | The method to classify exposure to sufficient or insufficient KT was described and applied uniformly across participants. | Y | KM measurement was performed with a periodontal probe in a valid and reproducible manner, by a single trained and calibrated examiner | Y | Confounding factors were identified. | Y | A univariate analysis was performed for all individual variables explored in the study, followed by a multivariate analysis for those with statistical significance | N | Participants were recalled for evaluation 6 or 8 years after implant placement; by this time, the outcome may already have occurred. | Y | The outcome was assessed in a valid and reproducible manner. There was a single trained and calibrated examiner; examinations were performed consistently using a periodontal probe and periapical radiography. | Y | Participants were followed for 6 or 8 years. | Y | Retrospective study; single recall at 6–8 years post-implant; non-returning participants excluded. | N | No imputation or sensitivity analysis was performed. The use of listwise deletion can introduce bias if missingness is related to the exposure or outcome. | Y | Statistical tests were appropriate for the analyses |
| Bozkurt2023 | U | All participants were from the same clinical setting, but the study does not present baseline comparability between KM vs non-KM groups (e.g., age, implant type, history of periodontitis). | Y | The exposure was measured in the same way for all patients | Y | KM measurement was performed with a periodontal probe in a valid and reproducible manner, by a single trained and calibrated examiner | Y | Confounders were identified. Exclusion of smokers, systemic disease; data collected on age, sex, implant brand, type of restoration, implant location, and time in function | N | Design-level control (restriction: no smokers/systemic disease; single surgeon). Statistical tests limited to Chi-square, Mann–Whitney, Fisher-Freeman–Halton (Statistical Analysis). No multivariate regression. | Y | Participants were followed from the time of implant placement | Y | The outcome was assessed in a valid and reproducible manner. There was a single trained and calibrated examiner; examinations were performed consistently using a periodontal probe and periapical radiography. | Y | Participants were followed from 12 to 46 months. | U | The study does not describe whether the follow-ups were complete. | N | No imputation or sensitivity analyses. | N | No regression analysis to control for confounders. |
| Costa2022 | Y | All participants were drawn from the same clinical cohort (UFMG), and from a previous cross-sectional study. | Y | The exposure was measured in the same way for all patients | Y | KM measurement was performed with a periodontal probe in a valid and reproducible manner, by two trained and calibrated examiners. | Y | The possible confounding factors were identified. | Y | A univariate analysis was performed for all individual variables explored in the study, followed by a multivariable logistic-regression models. | Y | Participants were followed from the time of implant placement. At T1 (entry into the PIMT program) all participants were clinically healthy or stabilized. | Y | The outcome was assessed in a valid and reproducible manner. Examiners were trained and calibrated; examinations were performed consistently using a periodontal probe and periapical radiography. | Y | Participants were followed for 11 years. | N | The sample of the initial study consisted of 80 participants. In this study, with a follow up of 11 years, 51 participants were analyzed and 26 excluded. | N | No imputation or weighting for missing records described. | Y | Statistical tests were appropriate for the analyses |
| Felice2023 | Y | Same population for both groups | Y | The exposure was measured in the same way for all patients | Y | KM measurement was performed with a periodontal probe in a valid and reproducible manner | Y | Confounding factors were identified and listed during the study. | Y | Multilevel logistic and linear regression model adjusted for confounders | Y | Participants were followed from the time of implant placement | Y | The outcome was assessed in a valid and reproducible manner; examinations were performed consistently using a periodontal probe and periapical radiography. | Y | Participants were followed from 1 to 8 years. | U | Retrospective, dropouts before 1 year were excluded; numbers before/after screening reported, but individual loss reasons not detailed | U | No imputation or sensitivity analysis described for excluded cases. | Y | Statistical tests were appropriate for the analyses |
| Fernandes2023 | U | The study does not present comparability between KT groups as regards variables (e.g., age, sex, site, prosthesis variables) | Y | The exposure was measured in the same way for all patients | Y | KM measurement was performed with a periodontal probe in a valid and reproducible manner, by two trained and calibrated examiners. | Y | Confounding factors were identified and listed during the study. | N | No matching, stratification, or regression adjustment to address confounding in estimating a KT effec | N | Participants were recalled for evaluation 2 years after implant placement; by this time, the outcome may already have occurred. | Y | The outcome was assessed in a valid and reproducible manner. There were two trained and calibrated examiners; examinations were performed consistently using a periodontal probe and periapical radiography. | Y | Participants were followed for 2 years. | U | The variation in follow-up time is very large (between 2 and 6 years), therefore in this context it is not possible to determine what would constitute a complete follow-up. | N | Unequal follow-up (2-6 years). No analytic strategy (e.g., survival rates over time, adjusted rates) is presented. | N | No multivariate analysis; no time-to-event analysis. |
| Mancini2024 | U | All patients belonged to the same source population (single university clinic, standardized follow-up), but the study does not present a baseline comparison of KM vs. no-KM groups. We therefore cannot confirm whether the groups were similar | Y | The exposure was measured in the same way for all patients | Y | KM measurement was performed with a periodontal probe in a valid and reproducible manner, by a single trained and calibrated examiner | Y | Confounding factors were identified and listed in a table. | Y | Logistic regression was used for the analysis of the variables. | Y | Participants were followed from the time of implant placement | Y | The outcome was assessed in a valid and reproducible manner. There was a single trained and calibrated examiner; examinations were performed consistently using a periodontal probe and periapical radiography. | Y | Participants were followed for 10 years. | U | The study does not describe whether the follow-ups were complete. Completeness and reasons for loss are not detailed | N | Exclusion is not a bias-mitigation strategy; no analytical handling of missingness is described | Y | Statistical tests were appropriate for the analyses |
| Nícoli2024 | U | In this SR, the exposure groups are defined according to keratinized mucosa (KM) width. However, the article does not provide a baseline table comparing KM vs no KM groups as regards patient characteristics (e.g., age, gender, smoking, systemic conditions). So, we cannot say that the two groups are similar . | Y | Exposures (e.g., KM width categories, connection type, prosthesis type) and clinical/radiographic factors were collected with the same procedures for all participants. The exposure is not peri-implant status. Peri-implant status is the outcome. | Y | KM measurement was performed with a periodontal probe in a valid and reproducible manner, by a single trained and calibrated examiner | Y | Confounding factors were identified and listed in a table. | Y | A univariate analysis was performed for all individual variables explored in the study, followed by a multivariate analysis for those with statistical significance | Y | Participants were followed from the time of implant placement | Y | The outcome was assessed in a valid and reproducible manner. There was a single trained and calibrated examiner; examinations were performed consistently using a periodontal probe and periapical radiography. | Y | Participants were followed for 12 months. | Y | Those who did not attend follow-up visits were excluded from the study. Further, there is a flow diagram, and the text details exclusions and missing radiographs at specific time points. | N | The study dealt with incomplete follow-up by excluding participants with missing data, but this approach does not constitute a bias-mitigation strategy as defined by JBI (e.g., imputation, adjustment for person-time, or sensitivity analyses). Actually, exclusion increases the risk of attrition bias. | Y | Statistical tests were appropriate for the analyses - multivariable modeling for confounding |
| Lv2024 | U | The paper does not present comparability between KTW (< 2 mm vs ≥ 2 mm) groups | Y | KTW was measured as part of a standardized peri-implant exam in all included cases. | U | KM exposure measurement was not described, only the periodontal and peri-implant status were reported. | Y | Confounding factors were identified and listed during the study. | Y | Participants with systemic diseases (diabetes) were excluded; multivariate analysis was used to assess other confounding factors. | Y | Participants were followed from the time of implant placement | Y | The outcome was assessed in a valid and reproducible manner, according to Mazza’s Bleeding Index and radiographic measurements using The Geometer’s Sketchpad software. | Y | Participants were followed for 4 years. | U | The study does not describe whether the follow-ups were complete. | U | No strategies (e.g., imputation, sensitivity analyses, person-time approaches) are described. | Y | Statistical tests were appropriate for the analyses |
| Poli2016 | Y | Same population for both groups | Y | The exposure was measured in the same way for all patients | Y | KM measurement was performed with a periodontal probe in a valid and reproducible manner, by a single trained and calibrated examiner | Y | Confounding factors were identified and listed during the study. | Y | Logistic regression was used for the analysis of the variables. | U | Participants were recalled for evaluation 12 years after implant placement; by this time, the outcome may already have occurred. | Y | The outcome was assessed in a valid and reproducible manner. There was a single trained and calibrated examiner; examinations were performed consistently using a periodontal probe and periapical radiography. | Y | Participants were followed for 12 years. | N | Retrospective, only surviving implants with records were analyzed; no reporting of attrition or missing-data reasons. | N | No imputation or weighting for missing records described. | Y | Statistical tests were appropriate for the analyses |
| Roccuzzo2025 | U | Baseline characteristics table stratified by KT vs AM is presented to confirm similarity. | Y | The visual assessment is considered appropriate in this study, as the predefined cutoff was the presence or complete absence of keratinized mucosa | Y | The measurement can be considered subjective, since it was performed visually only | Y | The possible confounding factors were identified. | U | The paper does not list which covariates entered the final adjusted model | Y | Participants were followed from the time of implant placement | Y | The outcome was assessed in a valid and reproducible manner. There was a single trained and calibrated examiner; examinations were performed consistently using a periodontal probe and periapical radiography. | Y | Participants were followed for 20 years. | Y | Those who did not attend follow-up visits were excluded from the study. | N | No mention of methods such as weighting, imputation, inverse-probability weighting, or sensitivity analyses to account for attrition. | Y | Statistical tests were appropriate for the analyses |
| Romandini2025 | U | Baseline characteristics table stratified by KT is presented to confirm similarity | Y | All exposures (periodontitis severity, smoking, sleep, etc.) were assessed at baseline using standardized examinations and structured questionnaires | N | The study does not describe how KM exposure was measured. | Y | The possible confounding factors were identified. | Y | Multilevel (mixed-effects) logistic regression was used, adjusting for multiple covariates | Y | Participants were followed from the time of implant placement | Y | The outcome was assessed in a valid and reproducible manner. Examiners were trained and calibrated; examinations were performed consistently using a periodontal probe and periapical radiography. | Y | Participants were followed for 3,9 years. | Y | Those who did not attend follow-up visits were excluded from the study. | NA | Those who did not attend follow-up visits were excluded from the study. | Y | Statistical tests were appropriate for the analyses |
| Ruiz-Romero2024 | U | The paper does not document baseline comparability between the KM < 2 mm and KM ≥ 2 mm exposure groups on key characteristics (e.g., age, smoking, periodontitis history) at cohort entry. | Y | The exposure was measured in the same way for all patients | Y | KM measurement was performed with a periodontal probe in a valid and reproducible manner | Y | Confounding factors were identified and listed during the study. | Y | A univariate analysis was performed for all individual variables explored in the study, followed by a multivariate analysis for those with statistical significance | N | Participants were recalled for evaluation 12 years after implant placement; by this time, the outcome may already have occurred. | Y | The outcome was assessed in a valid and reproducible manner; examinations were performed consistently using a periodontal probe and periapical radiography. | Y | Participants were followed for 12 years. | Y | Those who did not attend follow-up visits were excluded from the study. | N | No imputation or sensitivity analysis was performed. Exclusion can introduce bias if missingness is related to the exposure or outcome. | Y | Statistical tests were appropriate for the analyses |

"Legend: N, no; NA, Not applicable; U, unclear; Y, yes.

Q1: Were the two groups similar and recruited from the same population?

Q2: Were the exposures measured similarly to assign people to both exposed and unexposed groups?

Q3: Was the exposure measured in a valid and reliable way?

Q4: Were confounding factors identified?

Q5: Were strategies to deal with confounding factors stated?

Q6: Were the groups/participants free of the outcome at the start of the study (or at the moment of exposure)?

Q7: Were the outcomes measured in a valid and reliable way?

Q8: Was the follow up time reported and sufficient to be long enough for outcomes to occur?

Q9: Was follow up complete, and if not, were the reasons to loss to follow up described and explored?

Q10: Were strategies to address incomplete follow up utilized?

Q11: Was appropriate statistical analysis used?"

**Supplementary Table 4.** GRADE summary of findings

| **Certainty assessment** | | | | | | | | | | | | **№ of implants** | | **Effect** | | | **Certainty** |
| --- | --- | --- | --- | --- | --- | --- | --- | --- | --- | --- | --- | --- | --- | --- | --- | --- | --- |
| **№ of studies** | **Study design** | **Risk of bias** | | **Inconsistency** | | **Indirectness** | | **Imprecision** | | **Other considerations** | | **Keratinized mucosa present (> 0 mm or ≥ 2 mm)** | **Limited (< 2 mm) or absent keratinized mucosa** | **Relative (95% CI)** | | **Absolute (95% CI)** |  |
| **Risk of peri-implant mucositis between implants with keratinized mucosa present (> 0 mm) and implants without keratinized mucosa (0 mm)** | | | | | | | | | | | | | | |  |  |  |
| 5 | non-randomised studies | not serious | | serious^a^ | | not serious^e^ | | serious^f^ | | none | | 344/749 (45.9%) | 142/347 (40.9%) | **RR 0.92** (0.69 to 1.21) | | **33 fewer per 1.000** (from 127 fewer to 86 more) | ⨁◯◯◯ Very low^a^ |
| **Risk of peri-implant mucositis between implants with keratinized mucosa ≥ 2 mm and implants with keratinized mucosa < 2 mm** | | | | | | | | | | | | | | |  |  |  |
| 7 | non-randomised studies | not serious | | very serious^b^ | | not serious^e^ | | serious^f^ | | none | | 386/1290 (29.9%) | 220/609 (36.1%) | **RR 0.70** (0.41 to 1.20) | | **108 fewer per 1.000** (from 213 fewer to 72 more) | ⨁◯◯◯ Very low^b^ |
| **Risk of peri-implantitis between implants with keratinized mucosa present (> 0 mm) and implants without keratinized mucosa (0 mm)** | | | | | | | | | | | | | | |  |  |  |
| 5 | non-randomised studies | not serious^c^ | | not serious | | not serious^e^ | | serious^g^ | | none | | 122/820 (14.9%) | 43/390 (11.0%) | **RR 0.62** (0.42 to 0.91) | | **42 fewer per 1.000** (from 64 fewer to 10 fewer) | ⨁◯◯◯ Very low^c^ |
| **Risk of peri-implantitis between implants with ≥ 2 mm of keratinized mucosa and implants with < 2 mm of keratinized mucosa** | | | | | | | | | | | | | | |  |  |  |
| 9 | non-randomised studies | | not serious | | serious^d^ | | not serious^e^ | | not serious | | none | 192/1543 (12.4%) | 126/910 (13.8%) | **RR 0.56** (0.37 to 0.84) | | **61 fewer per 1.000** (from 87 fewer to 22 fewer) | ⨁⨁◯◯ Low^d^ |

**CI:** confidence interval; **RR:** risk ratio

#### Explanations

a. There was inconsistency among the studies, as evidenced by substantial heterogeneity (I² = 63%). Although confidence intervals were largely overlapping, the magnitude of unexplained variability across studies reduced confidence in the pooled estimate. Therefore, the certainty of the evidence was downgraded by one level due to inconsistency.

b. There was substantial inconsistency among the studies, as evidenced by considerable heterogeneity (I² = 93%). The wide dispersion of effect estimates indicates marked unexplained variability, suggesting that the pooled estimate may not adequately represent the individual study results. Therefore, the certainty of the evidence was downgraded by two levels due to inconsistency.

c. One study included in the analysis was assessed as having a high risk of bias; however, this study contributed minimal weight to the meta-analysis. In addition, sensitivity analyses confirmed that its exclusion did not substantially affect either the magnitude or the direction of the pooled effect. Consequently, the certainty of the evidence was not downgraded due to risk of bias.

d. There was inconsistency among the studies, as evidenced by substantial heterogeneity (I² = 69%,). Despite the presence of overlapping confidence intervals, the magnitude unexplained variability across studies reduced confidence in the pooled estimate. Therefore, the certainty of the evidence was downgraded by one level due to inconsistency.

e. No serious indirectness was identified, as the included studies directly addressed the population, exposure, comparison, and outcomes of interest defined in the review question.

f. Imprecision was rated down because the 95% confidence interval crossed the line of no effect and included both potential benefit and no clear effect, despite the relatively adequate number of events.

g. Imprecision was rated down because the total number of events appears to be below the optimal information size.
