# Supplementary material for: Keratinized Mucosa Width and Incidence of Peri‐Implant Diseases: A Systematic Review With Meta‐Analysis
Source: J Periodontal Res. 2026 May 14;61(5):452–72. doi: 10.1111/jre.70123 (PMC13378225; doi:10.1111/jre.70123)

**Supplementary Figure 1.** Forest plot for peri-implant mucositis comparing present (> 0 mm) vs. absent (0 mm) keratinized mucosa, with subgroup analysis by follow-up duration (≤5 years vs. > 5 years).


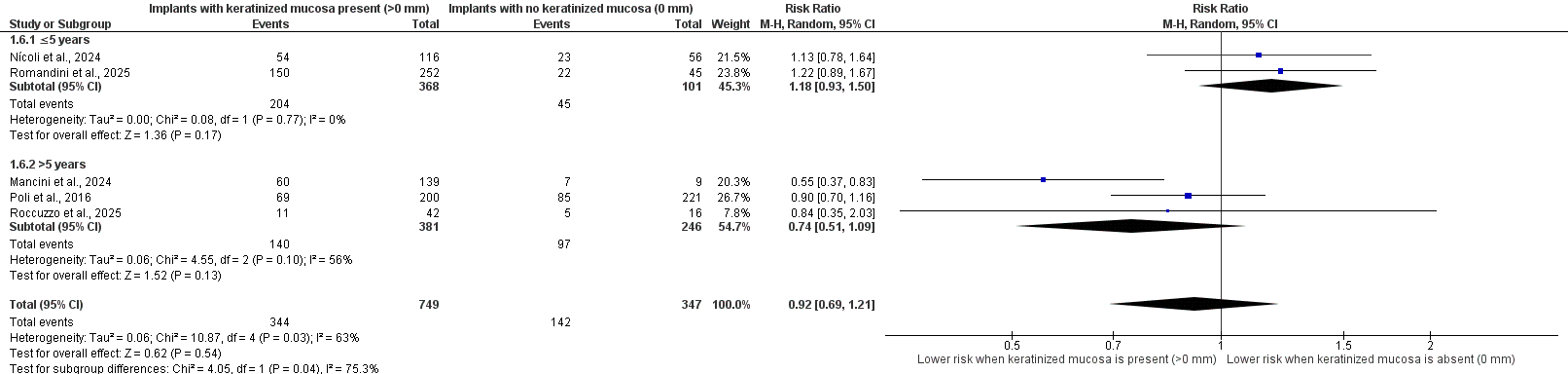


**Supplementary Figure 2.** Forest plot for peri-implant mucositis comparing ≥2 mm vs. < 2 mm of keratinized mucosa, with subgroup analysis by follow-up duration (≤5 years vs. > 5 years).


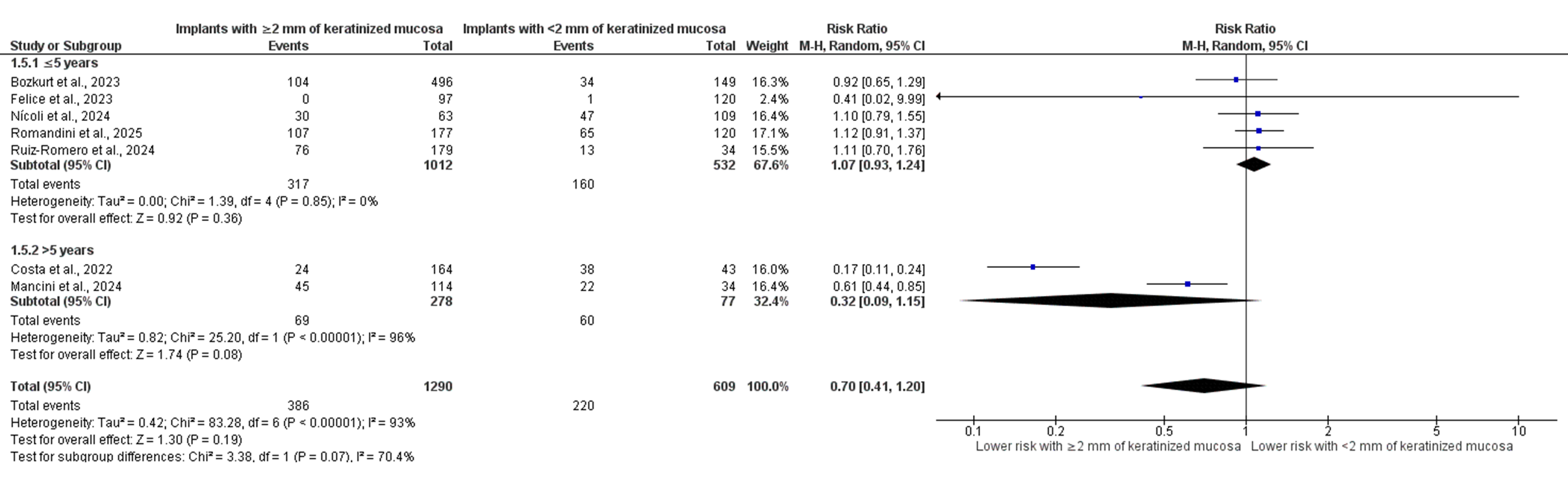


**Supplementary Figure 3.** Forest plot for peri-implantitis comparing present (> 0 mm) vs. absent (0 mm) keratinized mucosa, with subgroup analysis by follow-up duration (≤5 years vs. > 5 years).


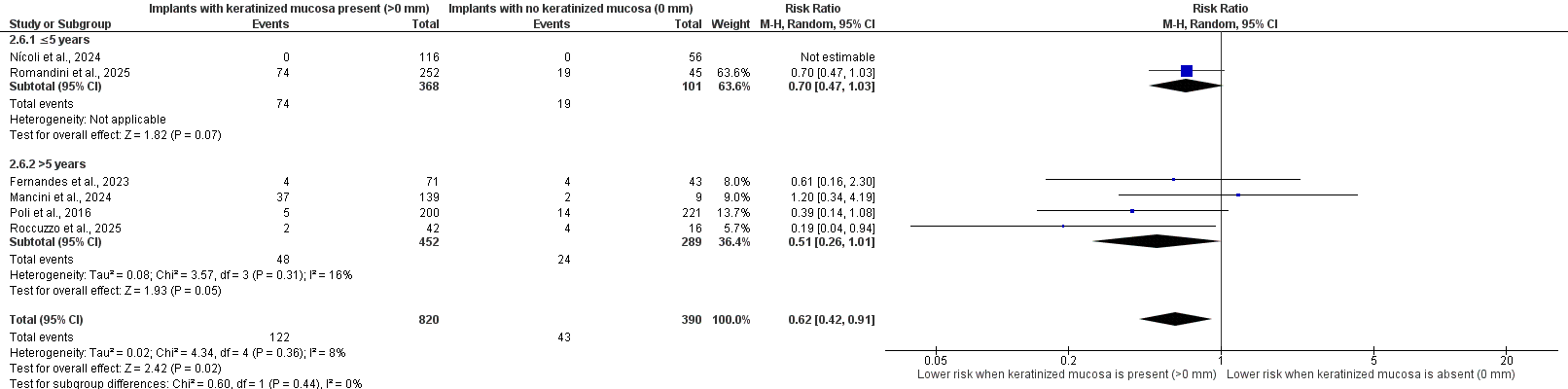


**Supplementary Figure 4.** Forest plot for peri-implant mucositis comparing ≥2 mm vs. < 2 mm of keratinized mucosa, with subgroup analysis by follow-up duration (≤5 years vs. > 5 years).


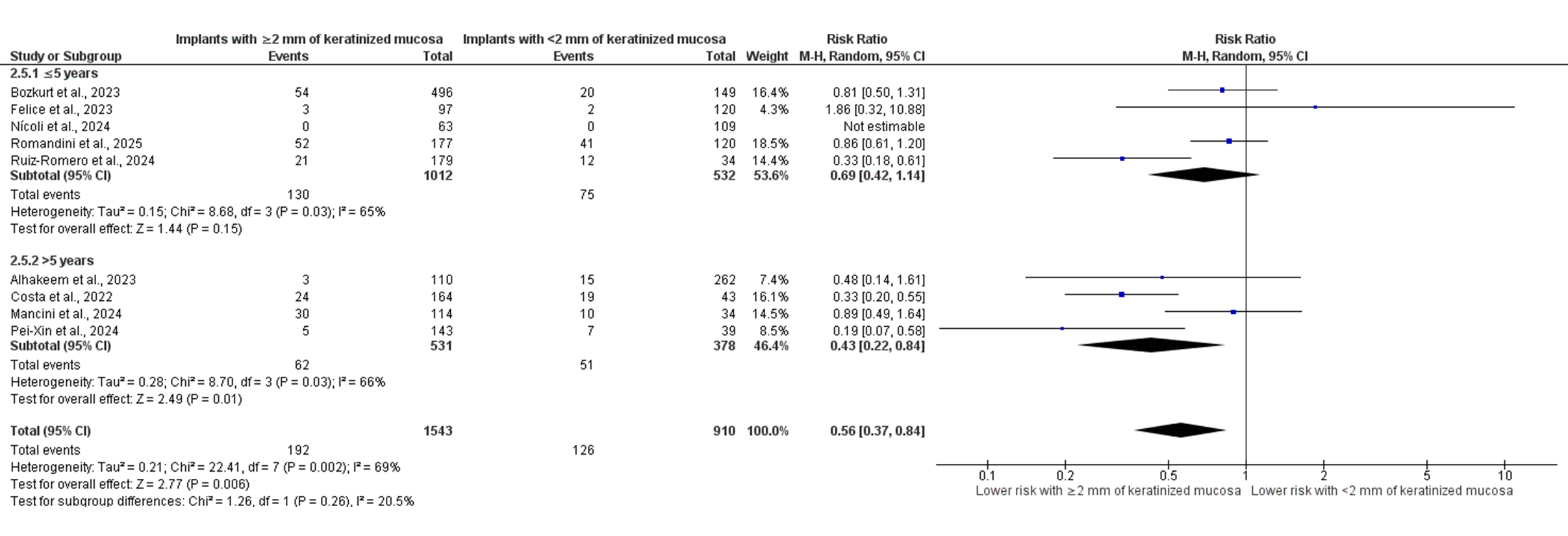


**Supplementary Figure 5.** Forest plot for peri-implantitis comparing present (> 0 mm) vs. absent (0 mm) keratinized mucosa, with subgroup analysis by peri-implantitis case definition thresholds (marginal bone loss and probing depth).


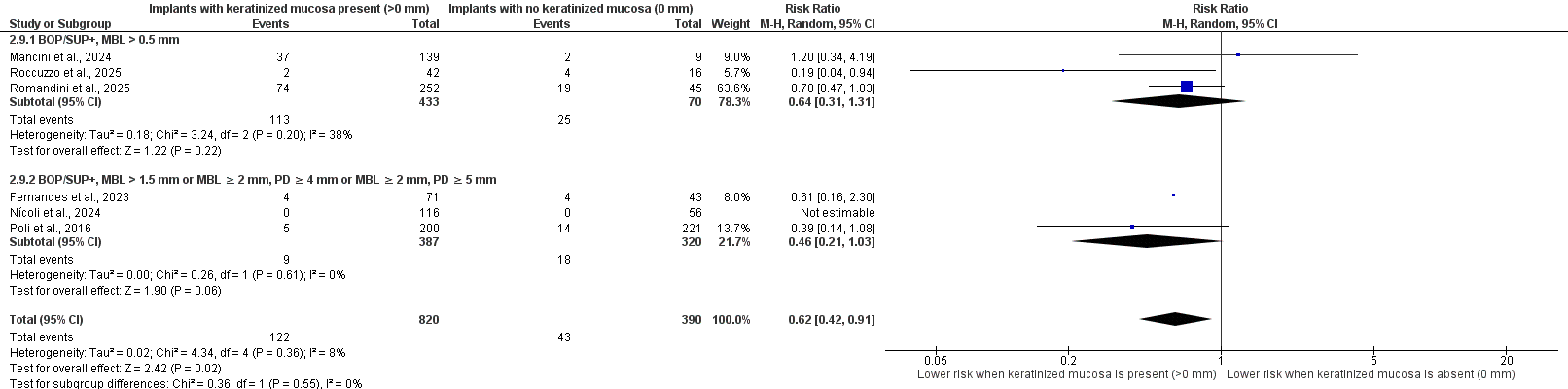


**Supplementary Figure 6.** Forest plot for peri-implantitis comparing 2 mm vs. < 2 mm of keratinized mucosa, with subgroup analysis by peri-implantitis case definition thresholds (marginal bone loss and probing depth).


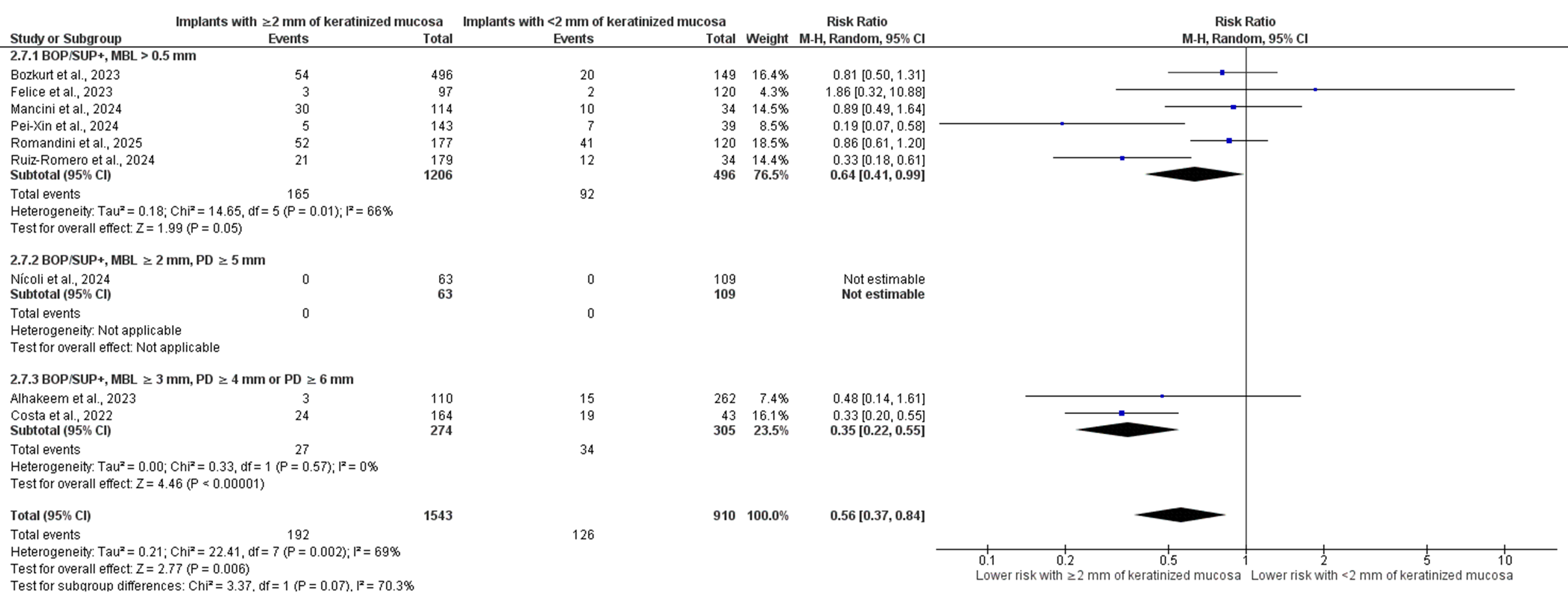

Supplement: Supplementary file 2 — Figure S1: Forest plot for peri‐implant mucositis comparing present (> 0 mm) vs. absent (0 mm) keratinized mucosa, with subgroup analysis by follow‐up duration (≤ 5 years vs. > 5 years). Figure S2: Forest plot for peri‐implant mucositis comparing ≥ 2 mm vs. < 2 mm of keratinized mucosa, with subgroup analysis by follow‐up duration (≤ 5 years vs. > 5 years). Figure S3: Forest plot for peri‐implantitis comparing present (> 0 mm) vs. absent (0 mm) keratinized mucosa, with subgroup analysis by follow‐up duration (≤ 5 years vs. > 5 years). Figure S4: Forest plot for peri‐implant mucositis comparing ≥ 2 mm vs. < 2 mm of keratinized mucosa, with subgroup analysis by follow‐up duration (≤ 5 years vs. > 5 years). Figure S5: Forest plot for peri‐implantitis comparing present (> 0 mm) vs. absent (0 mm) keratinized mucosa, with subgroup analysis by peri‐implantitis case definition thresholds (marginal bone loss and probing depth). Figure S6: Forest plot for peri‐implantitis comparing 2 mm vs. < 2 mm of keratinized mucosa, with subgroup analysis by peri‐implantitis case definition thresholds (marginal bone loss and probing depth). [file JRE-61-452-s001.docx]
